# Effects of different peripheral fatigue protocol on lower limb biomechanical changes during landing and its impact on the risk of anterior cruciate ligament injury: a systematic review

**DOI:** 10.3389/fbioe.2025.1587573

**Published:** 2025-06-03

**Authors:** Zhanyang He, Gang Sun, Houwei Zhu, Binyong Ye, Zhe Zheng, Xiaolong He, Huiju Pan

**Affiliations:** College of Physical Education and Health Sciences, Zhejiang Normal University, Jinhua, China

**Keywords:** fatigue, anterior cruciate ligament, neuromuscular, landing, biomechanics

## Abstract

Previous research suggests that although fatigue accumulates during competitions and at season’s end, anterior cruciate ligament (ACL) injuries do not significantly increase, with peripheral fatigue-induced muscle weakness potentially playing a key role. The aim of this study is to systematically review the effects of different peripheral fatigue interventions on biomechanical variables associated with ACL injury risk during landing tasks. A systematic search was conducted in five databases Web of Science, Scopus, PubMed, EBSCO, and Cochrane Library up to September 2024. The evidence classification system was used to grade the evidence on lower limb biomechanical changes. A total of 12 studies, involving 217 participants (105 males, 112 females), were included. These studies examined 9 peripheral fatigue protocols, 14 kinematic variables, and 16 kinetic variables. Among the 14 kinematic variables reviewed, strong evidence indicates increased knee internal rotation angles at peak vertical ground reaction force (vGRF) during landing tasks after the knee flexors and extensors peripheral fatigue protocols (Effect size = 0.24–0.68). For the 16 kinetic variables reviewed, strong evidence only suggests a reduction in peak vGRF during landing tasks after knee flexors and extensors peripheral fatigue protocols (Effect size = 0.12–0.32). In conclusion, we found that only two peripheral fatigue interventions were supported by evidence, while most kinematic and kinetic variables showed conflicting results, underscoring the need for further research. Such improvements will help clarify whether current neuromuscular ACL injury prevention programs need to be adapted to account for the biomechanical changes brought about by peripheral fatigue.

**Systematic Review Registration:** clinicaltrials.gov, identifier CRD42024593839.

## Graphical Abstract

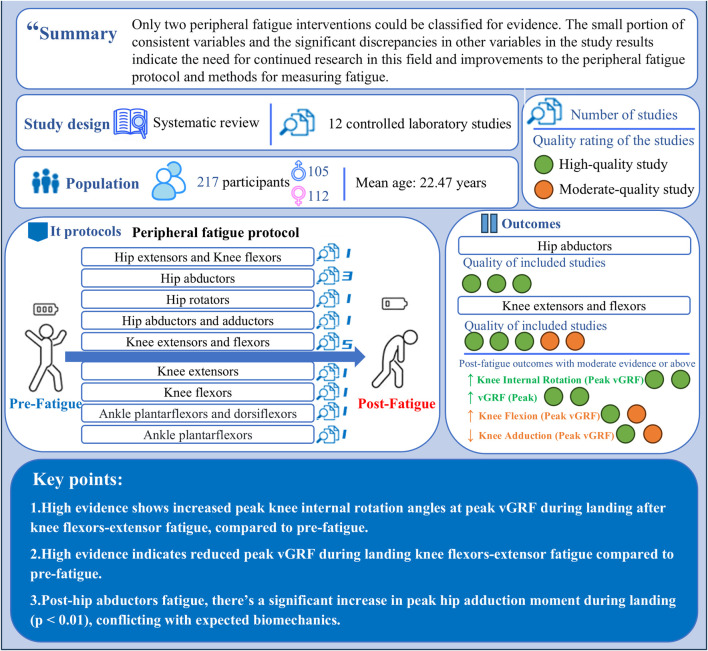

## 1 Introduction

Anterior cruciate ligament (ACL) injuries are among the most severe injuries in sports, significantly affecting an athlete’s performance upon return to competition and their long-term career (Davey et al., 2019). The majority of ACL injuries occur during non-contact actions such as landing, cutting, and pivoting (Achenbach et al., 2024). The incidence of ACL injuries and the associated rehabilitation costs are high, with approximately 100,000 to 250,000 cases occurring annually in the United States alone (Lohmander et al., 2004; Myklebust and Bahr, 2005). The estimated cost per injury is about $17,000 (Hewett et al., 2010). Across the United States, the total cost of surgeries and rehabilitation for female ACL injuries is approximately $646 million annually (Hewett et al., 2010). ACL injuries typically occur during the deceleration phase of landing maneuvers, such as single-leg landing (SL) or bilateral jumping and landing. Over the past few decades, most research on predicting ACL injury risk has focused on biomechanical differences in executing vertical landing tasks (Hewett et al., 2005; Mancino et al., 2023; Quatman et al., 2012). This has led to the development of several theoretical paradigms on ACL injury mechanisms during landing, including four major theories: ligament dominance, quadriceps dominance, trunk dominance, and leg dominance (He et al., 2024; Hewett et al., 2010). These theories have reached a preliminary consensus that ACL injury mechanisms involve changes in one or more biomechanical parameters. Building on these paradigms, previous scholars have identified eight biomechanical variables associated with ACL injury risk during landing (in the horizontal and sagittal planes, kinetic and kinematic sagittal plane motion, impact loading, and trunk mechanic) (Chou et al., 2023; He et al., 2024). For instance, the sagittal plane mechanism of ACL injury during landing is associated with smaller knee and hip flexion angles, which are linked to a higher risk of ACL injury (Padua et al., 2009; Trigsted et al., 2017). Therefore, identifying the biomechanical changes in individuals’ ACL injury patterns and the factors influencing these changes is crucial for predicting ACL injury risk.

Analyzing the biomechanical alterations induced by fatigue constitutes a fundamental aspect of ACL injury prevention research (Benjaminse et al., 2019). Fatigue arises from a combination of central and peripheral mechanisms (Enoka and Duchateau, 2007; Smeets et al., 2019). Central fatigue refers to a reduced capacity for voluntary muscle activation, attributed to a combination of spinal and supraspinal factors. This may include suboptimal neural drive from the motor cortex (Taylor et al., 2006), reduced motor neuron firing rates, and spinal excitability inhibition caused by muscle afferent input (Gandevia, 2001). Peripheral fatigue is defined as the reduction in efficiency at the neuromuscular junction and the distal ends of the muscles, involving metabolic and biochemical changes within the muscles themselves (Korzeniewski, 2019). The accumulation of metabolic byproducts such as reactive oxygen species, inorganic phosphates, calcium ions, lactate, ADP, and magnesium, along with glycogen depletion, disrupts homeostasis (Myburgh, 2004). Peripheral fatigue primarily manifests as impaired muscle function (e.g., decreased muscle strength and endurance), whereas central fatigue affects the athlete’s subjective feelings and neural recruitment capacity (Tornero-Aguilera et al., 2022). Previous studies have indicated that fatigue may play a critical role in ACL injuries in ball sports (Benjaminse et al., 2019). However, the dominant type of fatigue that leads to ACL injuries during athletic activities remains unclear. Furthermore, earlier research commonly suggests that athletes who spend more time training or competing bear a greater workload and experience higher levels of fatigue compared to those who train or compete less (Bourne et al., 2019). Consequently, the dominant perspective suggests that the risk of ACL injury escalates as a game progresses. However, contrary to this prevailing belief, a meta-analysis by Doyle et al. revealed no significant differences in ACL injury rates between the first and second halves, between the first half and fourth quarter, or between the initial and latter halves of the season (Doyle, 2018). In the second half of a game, athletes, especially those on the losing team, often face cognitive impairments such as stress, anxiety, and distraction, which could exacerbate central fatigue (Mehta and Parasuraman, 2013; Van Cutsem et al., 2017). Despite this, current evidence suggests that the exacerbation of central fatigue in the second half does not increase ACL injury risk. Therefore, the role of peripheral fatigue, which may dominate during athletic performance, has likely been overlooked in previous studies. Moreover, the hypothesis proposed by Mair et al. aligns with our own: ACL injuries during athletic activity may be caused by peripheral fatigue mechanisms (or muscle strength decline) (Mair et al., 1996). Such peripheral fatigue leads to a decrease in overall or localized muscle strength, which, in turn, causes harmful kinematic and kinetic changes in the hip, knee, and ankle joints, increasing the risk of ACL injury (Asaeda et al., 2024; Benjaminse et al., 2007; Kim et al., 2021).

Previous systematic reviews and meta-analyses have attempted to explore the relationship between fatigue and ACL injury-related biomechanical variables (Barber-Westin and Noyes, 2017; Benjaminse et al., 2019). However, none of these studies have found compelling evidence to confirm a definitive link between fatigue and ACL injuries (Barber-Westin and Noyes, 2017; Benjaminse et al., 2019). A common feature of these systematic reviews is that they include studies involving both central and peripheral fatigue induction, as well as those focusing exclusively on peripheral fatigue. The differences between fatigue types—and even variations in fatigue induction protocols (e.g., some protocols induce fatigue through isokinetic muscle contractions, while others use sport-specific fatigue tasks)—lead to significantly different biomechanical outcomes during landing (Barber-Westin and Noyes, 2017). This suggests that the mixed analysis of various types of fatigue in previous reviews may be a key reason for the lack of a clear relationship between fatigue and injury risk. Furthermore, Bourne et al. (2019) pointed out that no consistent effects have been observed on lower limb kinematic or kinetic variables under the fatigue protocols currently published (Bourne et al., 2019). This inconsistency is largely due to the broad definition of fatigue and the diverse forms of interventions, leading to high heterogeneity across studies, particularly concerning peripheral fatigue protocols. Currently, peripheral fatigue induction is typically conducted under controlled laboratory conditions. Specific methods include using isokinetic strength testing machines to perform repeated isometric contractions on the targeted muscle groups (Higo and Kuruma, 2021; Thomas et al., 2010a), or conducting multiple repetitions of contractions on one or more muscle groups under fixed or no-load conditions (Klein et al., 2023; Patrek et al., 2011), until a significant decline in muscle strength occurs. Each study targets different muscle groups for fatigue induction, which often results in a lack of consistency in biomechanical outcomes, making it difficult to identify the primary factor responsible for increasing ACL injury risk. Therefore, there is a need for a systematic summary of the biomechanical differences in landing caused by interventions targeting different muscle groups.

The primary objective of this systematic review is to comprehensively summarize the effects of different peripheral fatigue interventions on kinematics and dynamics during landing tasks. The secondary objective is to critically examine how different peripheral fatigue induction protocols and definitions of fatigue (Measure of Fatigue) influence lower limb biomechanics during landing. These two objectives are established to determine whether adjustments should be made to ACL injury prevention training programs to mitigate the harmful effects of peripheral fatigue on lower limb dynamics and kinematics. This study hypothesizes that, compared to the pre-fatigue, the execution of a landing task following a hip abductors fatigue protocol will result in a greater knee abduction angle. Furthermore, we hypothesize that even when the same fatigue protocol is applied to the same muscle group, individual biomechanical variables may exhibit conflicting changes after landing.

## 2 Methods

This review was conducted following the PRISMA guidelines for systematic reviews and meta-analyses. It has been pre-registered in the international prospective systematic review registry. PROSPERO registration number: CRD42024593839.

### 2.1 Data sources and search strategy

A systematic search was conducted in September 2024 across five databases: Web of Science, Scopus, PubMed, EBSCO, and the Cochrane Library. Within each group of keywords, Boolean operators “OR” were used for combinations, and “AND” was used to combine different groups of keywords within each database. No restrictions were placed on publication dates. The full search strategy across different databases is provided in the Supplementary Material (Supplementary Material S1).

### 2.2 Inclusion criteria

The search results were independently screened by two reviewers (ZH and HZ) by pre-specified inclusion and exclusion criteria. Any disagreements were resolved through consultation with the corresponding author (HP).

Studies meeting the following criteria, based on the PICOS framework, were included: **P**: Healthy adult participants (Age >18 years); **I**: Participants performing landing tasks with peripheral fatigue induction as an intervention; **C**: Comparison between pre- and post-fatigue intervention outcomes; **O**: Reported outcome measures related to sports biomechanics variables (e.g., ground reaction force, joint angles, joint moments); **S**: Controlled laboratory studies. Studies that do not meet the inclusion criteria will be excluded.

### 2.3 Data extraction and simplification

Titles and abstracts were screened by two independent reviewers, with any discrepancies resolved through consultation with a third reviewer. If eligibility could not be determined from the title and abstract, the full text was obtained and reviewed. Cohen’s kappa coefficient (κ) and consistency percentage were used to assess inter-rater agreement. The interpretation of κ was based on Landis and Koch’s standards: values less than 0 indicate no agreement, 0–0.20 indicate slight agreement, 0.21–0.40 indicate fair agreement, 0.41–0.60 indicate moderate agreement, 0.61–0.80 indicate substantial agreement, and 0.81–1 indicate almost perfect agreement (Landis and Koch, 1977). Full texts of all studies that met the inclusion criteria were retrieved, and the following data were extracted: (1) Authors’ names and publication year; (2) Sample size, gender, age, height, and weight of participants; (3) Type of sport and level of participation; (4) Details of the landing tasks used; (5) Fatigue induction protocols and measures of fatigue; (6) Biomechanical variable outcomes, including means, standard deviations, p-values (e.g., normalized ground reaction forces, joint angles, normalized joint moments), and their units of measurement.

### 2.4 Assessment and analysis of study quality

The methodological quality of the included studies was assessed using the Downs and Black checklist (Downs and Black, 1998) (Supplementary Table S2). The original checklist comprises 27 items covering key methodological aspects such as external validity, internal validity (including bias and confounding variables), and statistical power. It has demonstrated strong psychometric properties, including high criterion validity (*r* = 0.90), internal consistency (KR-20 = 0.89), test-retest reliability (*r* = 0.88), and inter-rater reliability (*r* = 0.75). For this study, we utilized a modified version with 17 items (Downs and Black, 1998). To reduce subjectivity in the interpretation of the original items, we adopted the method modified by Ramachandran et al. (2024). The original scoring scale for power-related items (0–5) was simplified to a binary format, with each question scored as 0 (No) or 1 (Yes). An exception was Item 20, which used a three-point scale: 0 (No), 1 (Partial), or 2 (Yes). A score of 2 was assigned if the study reported accuracy and provided a clear methodological description, while a score of 1 was given if only the methodology was described. These adjustments resulted in a maximum possible score of 18 for the evaluated studies. Scores obtained using the Downs and Black checklist were converted into percentage scores, and studies were classified as high quality (≥71%), moderate quality (51%–70%), or low quality (≤50%) (Kennelly, 2010). Two authors independently assessed the quality of the included studies, and any discrepancies were resolved through consultation with the corresponding author.

### 2.5 Strength of evidence synthesis

The included studies exhibited variations in landing tasks, fatigue induction protocols, and fatigue definitions, preventing a quantitative analysis. To evaluate the strength of evidence on biomechanical parameters associated with ACL injury before and after fatigue interventions, we applied the classification system by van Tulder et al. (2003):

**Strong evidence**: Consistent findings across a minimum of two high-quality studies.
**Moderate evidence**: Consistent findings across multiple studies, including at least one high-quality study.
**Limited evidence**: One high-quality study or multiple moderate- or low-quality studies.
**Very limited evidence**: One moderate- or low-quality study.
**Conflicting evidence**: Inconsistent findings across multiple studies.

To determine result consistency, all studies reporting specific kinematic or dynamic factors were included, irrespective of quality. The directionality of outcomes was assessed based on the mean values of biomechanical variables before and after fatigue induction and their statistical significance (p < 0.05). Consequently, the qualitative results were classified into four scenarios: increase, decrease, conflict, or no change.

## 3 Results

### 3.1 Search results and screening

**FIGURE 1:**
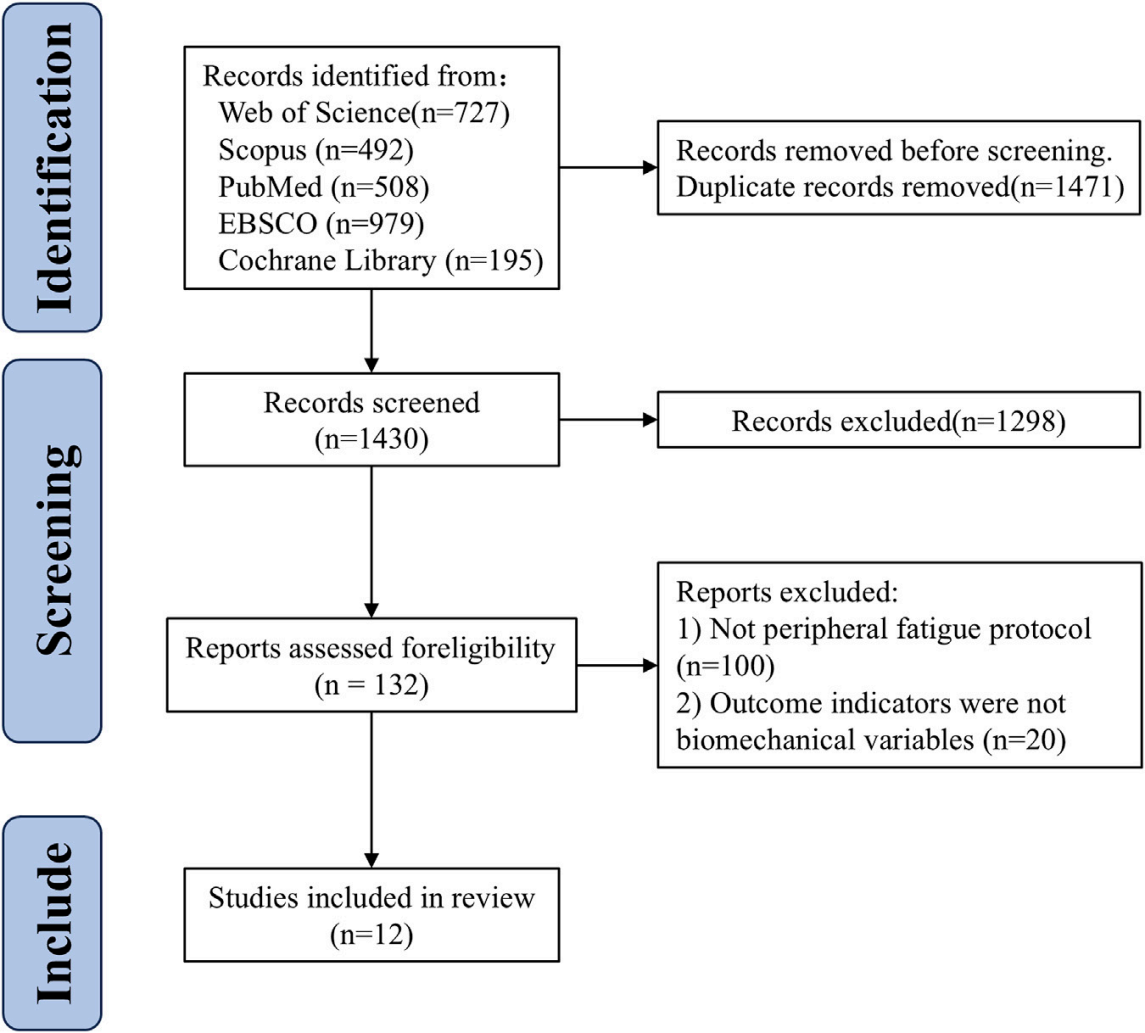
Flow chart of systematic reviews and meta-analyses.

A total of 2,901 studies were identified through five electronic databases: Web of Science, PubMed, Scopus, EBSCO, and the Cochrane Library. After removing duplicates, 1,430 studies remained. The title and abstract screening conducted by two reviewers excluded 1,298 studies (consistency rate = 99.61%, κ = 0.98), and 132 studies were assessed for eligibility through full-text review. Following this review, 120 studies were excluded, leaving 12 studies included in the review (consistency rate = 100%, κ = 1). A detailed exclusion process at each stage is shown in Figure 1.

### 3.2 Characteristics of the studies

This review included a total of 217 participants (105 males and 112 females). According to the data from the 12 studies, the average age of male participants was 22.47 ± 2.86 years, average height was 177.51 ± 7.30 cm, and average weight was 72.46 ± 11.05 kg. For female participants, the average age was 21.48 ± 2.07 years, average height was 167.53 ± 6.81 cm, and average weight was 60.15 ± 8.72 kg. Among the included studies, five studies enrolled healthy adults participating in recreational physical activities (Asaeda et al., 2024; Carcia et al., 2005; Klein et al., 2023; Thomas et al., 2010a; Thomas et al., 2010b), two studies included university physical education students (Kellis and Kouvelioti, 2007; Kim et al., 2017), two studies included healthy and physically active adults (Gehring et al., 2008; Kim et al., 2021), one study included NCAA Division III physically active female athletes (Patrek et al., 2011), and two studies did not report the participants’ activity levels (Higo and Kuruma, 2021; Wikstrom et al., 2004) (Table 1).

**TABLE 1:** Study characteristics and participant demographics.

Study (year)	Study design	Sample	Age (year)	Height (cm)	Weight (kg)	Sport types	Train level
Klein et al. (2023)	Controlled laboratory study	M7 F9	M21.3 ± 2.6 F22.3 ± 3.4	M 177.4 ± 8.8 F 169.2 ± 6.9	M 79.1 ± 11.9 F 66.6 ± 14.4	healthy, recreationally active university students	Recreational
Thomas et al. (2010a)	Controlled laboratory study	M13 F12	M20.31 ± 0.85 F20.33 ± 1.33	M180 ± 1 F170 ± 1	M 76.0 ± 8.9 F 58.3 ± 7.7	healthy recreational volunteers	Recreational
Higo and Kuruma, 2021	Controlled laboratory study	M12	23.3 ± 2.9	173.6 ± 5.8	65.9 ± 8.3	NR	NR
Patrek et al. (2011)	Descriptive laboratory study	F12	21.0 ± 1.3	167.9 ± 5.9	61.8 ± 8.4	physically active women	National Collegiate Athletic Association Division III
Wikstrom et al. (2004)	Controlled laboratory study	M8 F12	M21.8 ± 1.4 F22.2 ± 2.1	M180.6 ± 7.6 F169.3 ± 9.8	M 74.1 ± 13.0 F 62.5 ± 10.1	NR	NR
Carcia et al. (2005)	Controlled laboratory study	M10 F10	24 ± 2.8	174.2 ± 7.9	70.9 ± 12.7	recreationally active college-age students	Recreational
Asaeda et al. (2024)	Controlled laboratory study	M16	19.8 ± 0.9	173.5 ± 7.2	66.4 ± 11.7	healthy recreational adults	Recreational
Kim et al. (2021)	Controlled laboratory study	M5 F5	26.6 ± 1.35	175 ± 7	71.1 ± 14.1	healthy and physically active adults	NR
Kim et al. (2017)	Controlled laboratory study	M11 F13	M21.27 ± 2.24 F20.77 ± 1.01	M 179.68 ± 4.79 F 165.62 ± 6.51	M 72.20 ± 7.19 F 58.76 ± 6.58	physical education students	NR
Gehring et al. (2008)	Controlled laboratory study	M13 F13	M25.0 ± 2.4 F22.6 ± 1.5	M 180.5 ± 6.1 F 166.9 ± 5.7	M 74.8 ± 6.0 F 57.8 ± 4.2	physically active male and female	NR
Kellis and Kouvelioti (2007)	Controlled laboratory study	M10 F10	M24.3 ± 1.25 F23.5 ± 1.43	M181.3 ± 8.27 F168.9 ± 8.38	M 79 ± 8.21 F 59.82 ± 6.25	physical education students	NR
Thomas et al. (2010b)	Controlled laboratory study	F16	18–22	57.92 ± 6.87	164 ± 5	recreationally active volunteers	Recreational

Abbreviations: M, male; F, female; NR, no report.

The studies included in this review evaluated biomechanical risk factors for ACL injury during landing tasks following peripheral fatigue protocols. A total of nine different peripheral fatigue interventions were used. One study applied fatigue interventions to the hip extensors and knee flexors; another study targeted the hip internal and external rotators for peripheral fatigue. Additionally, one study focused on fatigue of the hip abductors and adductors. One study focused on the knee extensors, while another targeted the knee flexors for fatigue intervention. Additionally, one study induced fatigue in the ankle dorsiflexors, while another concentrated on the ankle plantarflexors. Notably, three studies applied peripheral fatigue interventions to the hip abductors, while five studies focused on the fatigue of the knee extensors and flexors (Table 1).

Concerning the definition and measurement of fatigue, one study objectively assessed fatigue by applying 20%, 30%, and 50% of maximal isometric voluntary contraction (MIVC) to the target muscle groups, while another employed the Borg Scale (≥19 or 13–14) to evaluate subjective fatigue. A third study measured objective fatigue using electromyography (EMG) average frequency and MIVC, whereas another combined both MIVC and the Borg Scale to assess both subjective and objective fatigue. Additionally, one study defined fatigue as the point at which participants could no longer perform the selected load after repeated contractions (Table 2).

**TABLE 2:** Fatigue intervention protocols and experimental procedures.

Study (year)	Target areas for fatigue intervention	Task (height of landing)	Fatigue protocol	Measure of fatigue	Experimental protocol
Klein et al. (2023)	Hip extensors and knee flexors	single-leg drop-jump (height 0.3 m, the distance from the FP 0.15 m)	3 sets of gluteus-hamstring raises and Nordic hamstring curls	1.After 3 sets of each exercise, fatigue of the medial and lateral hamstrings was measured using a handheld dynamometer. 2. If the participant’s force output dropped by 20% from their MIVC, they were considered adequately fatigued. 3. If the 20% reduction was not achieved, the participant repeated 3 additional sets of the exercises.	3 single-leg drop-jumps, fatigue protocol, 3 single-leg drop-jumps
Thomas et al. (2010a)	Knee flexors and extensors	single-leg takeoff and landing (Hop distance was normalized to 1 × leg length)	Alternating Quadriceps and Hamstring maximum voluntary concentric contractions	Until the torque measured in both muscle gorups dropped below 50%	3hops, fatigue protocol, 3hops
Higo and Kuruma, 2021	Knee flexors and extensors	Single leg landing (height 0.3m, the distance from the FP 0.2 m)	Isokinetic knee extension/flexion movement with an angular velocity of 180°/s over a 90°–0° range of motion for 10 min.	Borg scale: Continuously displayed, target score of 13–14. Heart rate: Monitored to ensure it does not exceed 100 bpm. Respiratory rate: Monitored to ensure it does not exceed 25 breaths/min.	3 Single leg lands, fatigue protocol, 3 Single leg lands
Patrek et al. (2011)	Hip abductors	single-leg drop landing (height 0.4 m)	hip-abductor fatigue protocol consisting of repetitive side-lying hip abduction.	Borg scale≥19	5 single-leg drop landings, Fatigue protocol, 5 single-leg drop landings
Wikstrom et al. (2004)	Ankle dorsiflexors and plantarflexors	2-legged jumps equivalent to 50% of maximum jump height, followed by a single-leg landing (distance 0.7 m)	Isokinetic ankle plantar-flexion and dorsiflexion movement concentric Peak Torque at 30s^-1^ and 120s^-1^	less than 50% peak torque	3 jump-landings, fatigue protocol,3 jump-landings
Carcia et al. (2005)	Hip abductors	drop jump (height 0.3 m)	Isometric bilateral hip-abductor-fatigue protocol.	Inability to achieve 50% of their ipsilateral baseline force bilaterally for 2 trials.	3 drop jumps, fatigue protocol, 3 drop jumps
Asaeda et al. (2024)	Knee flexors and extensors/Hip abductor and adductors	one-leg jump landing (height 0.2 m)	Task 1: knee fatigue task protocol (isokinetic knee extension/flexion movement with an angular velocity set at 120°/s, completing 40 repetitions for each movement). Task 2: hip fatigue task protocol (isokinetic hip abduction/adduction movement with an angular velocity set at 60°/s, completing 40 repetitions for each movement.)	Objective fatigue was assessed by comparing muscle torque values from the first and last three attempts of the task. Subjective fatigue was evaluated using the Borg scale.	3 one-leg jump landings, fatigue protocol, 3 one-leg jump landings
Kim et al. (2021)	Hip abductors	single-leg landing (height 0.45 m)	Participants performed three sets of hip abduction exercises without additional weight at a rate of 60 repetitions per minute, until they could no longer achieve the target angle of 35° of hip abduction. A 2-min rest was provided between sets.	MVIC for 5 s using standard manual muscle testing positions with manual resistance. Target muscle EMG mean power frequency was calculated to assess whether the muscle frequency spectrum shifted to lower frequencies, thereby confirming fatigue.	3 single-leg landings, fatigue protocol, 3 single-leg landings
Kim et al. (2017)	Knee flexors and extensors	45° side step after jump landing (height 0.3 m and 0.4 m)	Isokinetic flexion/extension of the knee joint exercise ROM: from 90° of knee flexion to 0° of knee extension. Angular velocity: 60°/s	post-50%, and post-30% fatigue levels of the knee extension peak torque	5 45° side step after jump landings, 50% fatigue of knee extension peak torque,5 45° side step after jump landings,30% fatigue of knee extension peak torque, 5 45° side step after jump landings
Gehring et al. (2008)	Knee flexors and extensors	bilateral landing task (height 0.52 m)	sub-maximal fatigue protocol on a leg press weight machine. (Subjects performed knee flexion and extension (90° to full extension) using 50% of their maximum load (determined by a 1-repetition maximum test).)	Until the subjects could no longer perform the selected load, they were considered fatigued.	3 bilateral landings, fatigue protocol, 3 bilateral landings
Kellis and Kouvelioti (2007)	Knee extensors/Knee flexors	single-leg landings (height 0.3 m)	2 sets of consecutive concentric efforts of the knee extensors or flexor on a dynamometer	Until the subject could no longer produce 30% of the maximum moment	NR
Thomas et al. (2010b)	Hip rotators/Ankle plantarflexors	Single-leg drop landing (height 0.17 m)	Hip rotators fatigue protocol, Triceps surae fatigue protocol	when the first five maximum voluntary concentric contractions of any given set were performed 80% below the baseline peak torque measure.	3 single leg drop landings, fatigue protocol, 3 single leg drop landings

Abbreviations: NR, no report; FP, force platform; MIVC, maximal isometric voluntary contraction; EMG, electromyography.

Furthermore, the studies included in this review also assessed the biomechanical risk factors for ACL injury during various jumping tasks. Seven studies employed the SL task, one study used the single-leg takeoff and landing task, one study applied the SL drop jump, one study used the drop jump (DJ) task, one study performed the bilateral landing task, and one study used a 45° side-step after jump landing task.

### 3.3 Methodological quality

The overall methodological quality score of the included studies (mean ± SD) was 14 ± 1.58 points (76% ± 8.43%). Eight studies were rated as high quality, and four studies were rated as moderate quality. Detailed scores for each study are presented in Table 3.

**TABLE 3:** Quality rating of the included studies as per the Downs and Black checklist.

Study (year)	Reporting							External validity		Internal validity				Internal validity - confounding (selection bias)				Result		
	1	2	3	5	6	7	10	11	12	15	16	18	20	21	22	25	27	Total score	Overall rating	Quality
Klein et al. (2023)	1	1	1	1	1	1	1	1	0	0	1	1	2	1	0	1	0	14	78%	High
Thomas et al. (2010a)	1	1	1	1	1	1	1	1	0	0	1	1	2	0	0	1	1	14	78%	High
Higo and Kuruma, 2021	1	1	1	1	1	1	0	0	0	0	1	1	2	0	0	1	1	12	67%	Moderate
Patrek et al. (2011)	1	1	1	1	1	1	1	1	1	0	1	1	2	1	0	1	1	16	89%	High
Wikstrom et al. (2004)	1	1	1	1	1	1	1	1	0	0	1	1	1	0	0	1	0	12	67%	Moderate
Carcia et al. (2005)	1	1	1	1	1	1	1	1	0	0	1	1	1	1	0	1	0	13	72%	High
Asaeda et al. (2024)	1	1	1	1	1	1	1	1	1	0	1	1	2	1	1	1	1	17	94%	High
Kim et al. (2021)	1	1	1	1	1	1	1	1	0	0	1	1	2	0	0	1	1	14	78%	High
Kim et al. (2017)	1	1	1	1	1	1	1	1	0	0	1	1	2	1	0	1	0	14	78%	High
Gehring et al. (2008)	1	1	0	1	1	1	1	1	0	0	1	1	2	0	0	1	0	12	67%	Moderate
Kellis and Kouvelioti (2007)	1	1	0	1	1	1	1	1	0	0	1	1	2	0	0	1	0	12	67%	Moderate
Thomas et al. (2010a)	1	1	1	1	1	1	1	1	0	0	1	1	2	0	0	1	1	14	78%	High

### 3.4 Evidence synthesis strength

**FIGURE 2:**
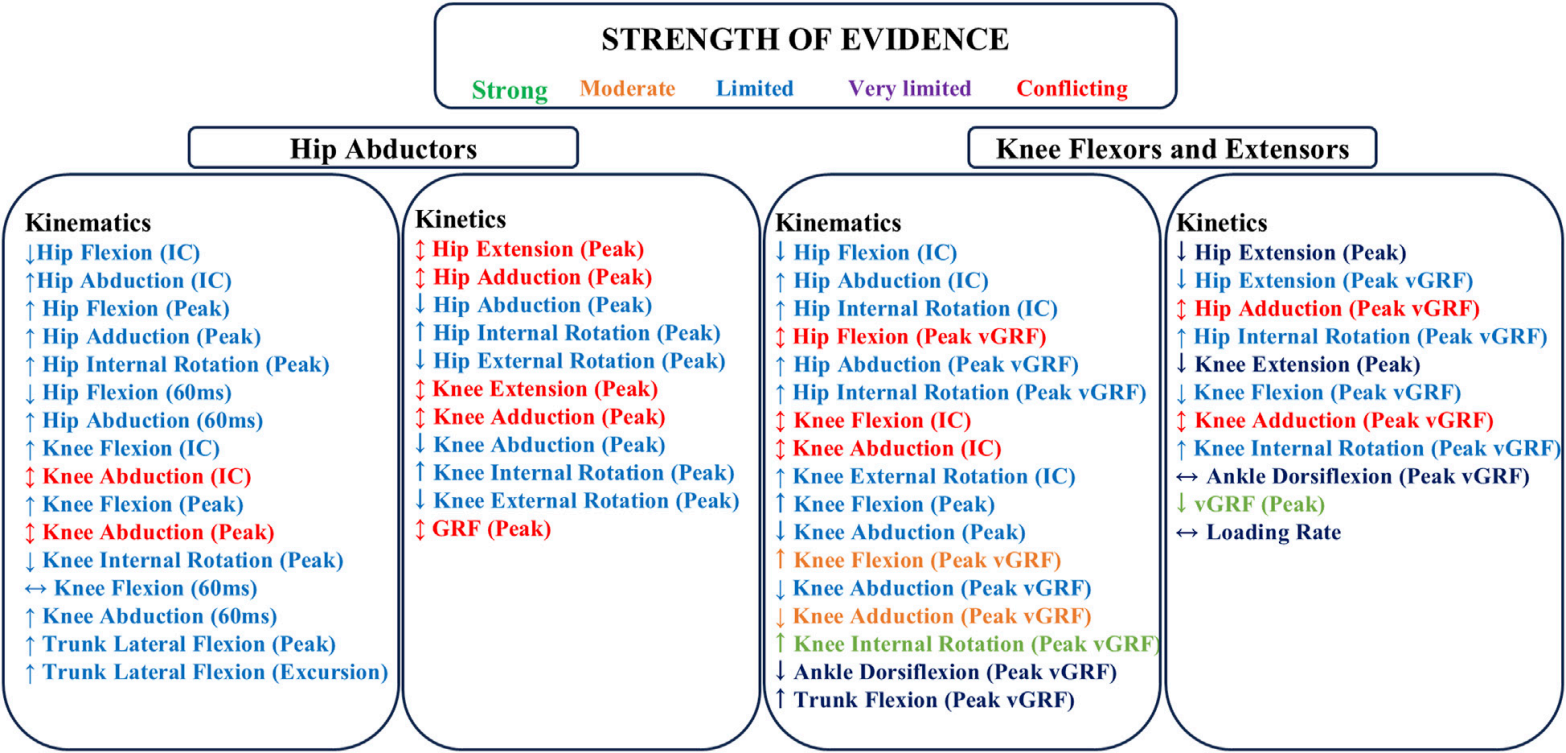
Summarize the strength of evidence for kinematic and kinetic variables pre- and post-fatigue of hip abductors, knee flexors and extensors. ↑: Parameter changes greater than pre-fatigue; ↔: Parameter changes similar to pre-fatigue; ↓: Parameter changes less than pre-fatigue; ↕: indicates conflicting evidence; IC: Initial Contact; vGRF: vertical Ground Reaction Force.

In the hip abductor fatigue protocol intervention, strong evidence was found indicating that compared to the pre-fatigue condition, post-fatigue participants showed an increased hip abduction angle at both the initial contact (IC) and peak moments during the landing task (Figure 2). The knee extensors and flexors fatigue protocol intervention provided strong evidence that post-fatigue, participants exhibited a greater knee internal rotation angle at peak vertical ground reaction force (vGRF) and a reduced peak vGRF compared to pre-fatigue. For all other kinematic and kinetic variables, the evidence strength ranged from moderate to conflicting, with some instances where definitive conclusions regarding biomechanical changes could not be drawn (Table 4 and 5).

**TABLE 4:** Kinematic variables of the lower limb in landing tasks across various conditions: A synthesis of included studies.

Study	Target areas for fatigue intervention	Phase of kinematic data	Unit of measure	Pre-fatigue	Post-fatigue	Study finding
Hip Flexion angle						
Klein et al. (2023)	Hip extensors and knee flexors	IC, Peak, Mean	Degree	IC:M:25.56 ± 3.6, F:20.83 ± 4.72; Peak: M:42.9 ± 9.25, F:40.09 ± 8.98; Mean: M:31.12 ± 7.29, F:29.16 ± 8.69	IC:M:23.06 ± 5.83, F:17.10 ± 7.29; Peak: M:40.37 ± 9.82, F:33.93 ± 11.21; Mean: M:28.6 ± 8.69, F:22.99 ± 10.65	IC: NS: P > 0.05 (↓); Peak: NS: P > 0.05 (↓); Mean: NS: P > 0.05 (↓)
Thomas et al. (2010a)	Knee flexors and extensors	IC, Peak vGRF	Degree	IC: M:26.1 ± 4.0, F:31.5 ± 8.3; Peak vGRF: M:27.6 ± 4.3, F:33.4 ± 9.8	IC: M:24.1 ± 7.7, F:31.6 ± 9.8; Peak vGRF: M25.0 ± 8.3, F33.0 ± 10.7	IC: NS: P > 0.05 (↓); Peak vGRF: NS: P > 0.05 (↓)
Higo and Kuruma (2021)	Knee flexors and extensors	Peak vGRF	Degree	Peak vGRF: 25.7 ± 6.7	Peak vGRF: 26.8 ± 8.4	Peak vGRF: NS: P > 0.05 (↑)
Patrek et al. (2011)	Hip abductors	IC, 60 ms after IC	Degree	IC: 2.7 ± 5.1; 60 ms after IC: 6.5 ± 6.1	IC:3.0 ± 5.0; 60 ms after IC: 5.8 ± 6.3	IC: NS: P > 0.05 (↓); 60 ms after IC: NS: P > 0.05 (↓)
Kim et al. (2021)	Hip abductors	Peak	Degree	Peak: 46.58 ± 9.54	Peak: 47.62 ± 9.42	Peak: NS: P > 0.05 (↑)
Kellis and Kouvelioti (2007)	Knee extensors	IC, Peak	Degree	IC: 2.75 ± 0.84; Peak: 32.00 ± 7.97	IC: 3.16 ± 1.01; Peak: 40.91 ± 9.83	IC: NS: P > 0.05 (↑); Peak: p < 0.05 (↑)
Kellis and Kouvelioti (2007)	Knee flexors	IC, Peak	Degree	IC: 2.82 ± 0.57; Peak:33.29 ± 5.93	IC:2.99 ± 0.95; Peak: 34.93 ± 9.56	IC: NS: P > 0.05 (↑); Peak: NS: P > 0.05 (↑)
Thomas et al. (2010b)	Hip rotators	IC, Peak	Degree	IC: 36.6 ± 6.2; Peak: 44.6 ± 6.3	IC: 37.0 ± 5.6; Peak: 45.7 ± 6.2	IC: NS: P > 0.05 (↓); Peak: NS: P > 0.05 (↑)
Thomas et al. (2010a)	Ankle plantarflexors	IC, Peak	Degree	IC: 36.8 ± 6.6; Peak: 47.0 ± 7.4	IC: 36.1 ± 7.0; Peak: 47.9 ± 7.9	IC: NS: P > 0.05 (↑); Peak: NS: P > 0.05 (↑)
Hip abduction angle						
Thomas et al. (2010a)	Knee flexors and extensors	IC	Degree	IC: M: −8.5 ± 3.9, F: −3.8 ± 4.1	IC: M: −9.3 ± 5.0, F: −5.2 ± 3.5	IC: NS: P > 0.05 (↑)
Patrek et al. (2011)	Hip abductors	IC, 60 ms after IC	Degree	IC: 9.9 ± 3.6; 60 ms after IC: 9.6 ± 4.2	IC: 10.7 ± 3.4; 60 ms after IC: 9.7 ± 4.3	IC: p < 0.05 (↑); 60 ms after IC: NS: P > 0.05 (↑)
Thomas et al. (2010b)	Hip rotators	IC, Peak	Degree	IC: 8.9 ± 9.5; Peak: 9.5 ± 9.2	IC: 8.5 ± 6.9; Peak: 8.9 ± 7.0	IC: NS: P > 0.05 (↓); Peak: NS: P > 0.05 (↓)
Thomas et al. (2010a)	Ankle plantarflexors	IC, Peak	Degree	IC: 8.5 ± 6.8; Peak: 9.1 ± 6.7	IC: 8.7 ± 6.6; Peak: 9.2 ± 6.9	IC: NS: P > 0.05 (↑), Peak: NS: P > 0.05 (↑)
Thomas et al. (2010a)	Knee flexors and extensors	Peak vGRF	Degree	Peak vGRF: M: −6.4 ± 4.2, F: −1.1 ± 5.3	Peak vGRF: M: −7.1 ± 5.4, F: −3.3 ± 2.4	Peak vGRF: NS: P > 0.05 (↑)
Hip adduction angle						
Asaeda et al. (2024)	Knee flexors and extensors	Peak vGRF	Degree	Peak vGRF: 0.087 ± 5.615	Peak vGRF: 0.153 ± 4.912	Peak vGRF: NS: P > 0.05 (↑)
Asaeda et al. (2024)	Hip abductor and adductors	Peak vGRF	Degree	Peak vGRF: 1.307 ± 5.484	Peak vGRF: 0.709 ± 5.596	Peak vGRF: NS: P > 0.05 (↓)
Kim et al. (2021)	Hip abductors	Peak	Degree	Peak: 2.08 ± 8.72	Peak: 3.22 ± 9.62	Peak: NS: P > 0.05 (↑)
Hip internal rotation angle						
Thomas et al. (2010a)	Knee flexors and extensors	IC, Peak vGRF	Degree	IC: M:1.9 ± 7.1, F: −1.5 ± 7.6; Peak vGRF:M: 3.4 ± 6.6, F: 0.6 ± 8.7	IC: M:5.1 ± 7.6, F:1.8 ± 6.2; Peak vGRF: M: 4.4 ± 8.6, F: 2.9 ± 7.4	IC: p < 0.05 (↑); Peak vGRF: NS: P > 0.05 (↑)
Asaeda et al. (2024)	Knee flexors and extensors	Peak vGRF	Degree	Peak vGRF: 20.00 ± 10.47	Peak vGRF: 18.19 ± 13.07	Peak vGRF: NS: P > 0.05 (↓)
Asaeda et al. (2024)	Hip abductor and adductors	Peak vGRF	Degree	Peak vGRF: 18.03 ± 9.528	Peak vGRF: 16.20 ± 10.13	Peak vGRF: NS: P > 0.05 (↓)
Kim et al. (2021)	Hip abductors	Peak	Degree	Peak: 1.30 ± 5.98	Peak: 1.39 ± 7.58	Peak: NS: P > 0.05 (↑)
Thomas et al. (2010a)	Hip rotators	IC, Peak	Degree	IC: 4.7 ± 7.0; Peak: 9.8 ± 7.5	IC: 7.8 ± 9.5; Peak: 12.4 ± 9.4	IC: p < 0.05 (↑); Peak: p < 0.05 (↑)
Thomas et al. (2010a)	Ankle plantarflexors	IC, Peak	Degree	IC: 4.3 ± 6.8; Peak: 8.6 ± 7.2	IC: 5.0 ± 8.7; Peak: 9.2 ± 8.7	IC: NS: P > 0.05 (↑), Peak: NS: P > 0.05 (↑)
Knee flexion angle						
Thomas et al. (2010a)	Knee flexors and extensors	IC, Peak vGRF	Degree	IC: M: −11.9 ± 4.4, F: −14.9 ± 6.1; Peak vGRF: M: −22.9 ± 5.3, F: −28.1 ± 11.1	IC: M: −5.5 ± 5.6, F: −9.5 ± 6.6; Peak vGRF: M: −15.6 ± 5.5, F: −23.0 ± 10.3	IC: p < 0.05 (↓); Peak vGRF: NS: P > 0.05 (↑)
Higo and Kuruma (2021)	Knee flexors and extensors	Peak vGRF	Degree	Peak vGRF: 37.6 ± 6.2	Peak vGRF: 38.0 ± 6.1	Peak vGRF: NS: P > 0.05 (↑)
Patrek et al. (2011)	Hip abductors	IC, 60 ms after IC	Degree	IC: 1.8 ± 3.9; 60 ms after IC: 20.8 ± 4.6	IC: 2.3 ± 4.8; 60 ms after IC: 20.8 ± 5.3	IC: NS: P > 0.05 (↑); 60 ms after IC: NS: P > 0.05 (↔)
Wikstrom et al. (2004)	Ankle dorsiflexors and plantarflexors	Peak	Degree	Peak: 130.0 ± 9.0	Peak: 129.0 ± 8.0	Peak: NS: P > 0.05 (↑)
Kim et al. (2021)	Hip abductors	Peak	Degree	Peak: 65.54 ± 7.68	Peak: 66.74 ± 7.21	Peak: NS: P > 0.05 (↑)
Gehring et al. (2008)	Knee flexors and extensors	IC, Peak	Degree	IC: M: 15.9 ± 1.7, F: 15.9 ± 1.7; Peak: M: 71.8 ± 3.1, F: 87.6 ± 2.7	IC: M: 16.7 ± 1.4, F: 16.5 ± 1.5; Peak: M: 76.3 ± 3.7, F: 90.0 ± 2.0	IC: NS: P > 0.05 (↑); Peak: NS: P > 0.05 (↑)
Kellis and Kouvelioti (2007)	Knee extensors	IC, Peak	Degree	IC: 10.62 ± 2.58; Peak: 47.37 ± 9.35	IC: 18.50 ± 4.09; Peak: 61.46 ± 9.77	IC: p < 0.05 (↑); Peak: NS: P > 0.05 (↑)
Kellis and Kouvelioti (2007)	Knee flexors	IC, Peak	Degree	IC: 9.75 ± 2.29; Peak: 47.17 ± 9.77	IC: 15.00 ± 3.74; Peak: 57.84 ± 11.68	IC: p < 0.05 (↑); Peak: p < 0.05 (↑)
Thomas et al. (2010a)	Hip rotators	IC, Peak	Degree	IC: 15.4 ± 4.7; Peak: 55.5 ± 6.9	IC: 13.8 ± 5.8; Peak: 56.5 ± 7.5	IC: NS: P > 0.05 (↓); Peak: NS: P > 0.05 (↑)
Thomas et al. (2010a)	Ankle plantarflexors	IC, Peak	Degree	IC: 14.4 ± 5.5; Peak: 58.0 ± 8.2	IC: 12.4 ± 5.7; Peak: 55.4 ± 6.3	IC: p < 0.05 (↓); Peak: NS: P > 0.05 (↓)
Knee abduction angle						
Thomas et al. (2010a)	Knee flexors and extensors	IC, Peak vGRF	Degree	IC: M: −1.2 ± 1.7, F: −2.0 ± 2.6; Peak vGRF: M: −1.2 ± 2.6, F: −3.1 ± 6.0	IC: M: −1.7 ± 3.7, F: −1.4 ± 2.8; Peak vGRF: M: −1.7 ± 3.7, F: −2.1 ± 4.9	IC: NS: P > 0.05 (↕); Peak vGRF: NS: P > 0.05 (↕)
Patrek et al. (2011)	Hip abductors	IC, 60 ms after IC	Degree	IC: 0.4 ± 2.1; 60 ms after IC: 0.6 ± 3.5	IC: 0.1 ± 2.1; 60 ms after IC: 0.2 ± 3.6	IC: p < 0.05 (↓); 60 ms after IC: p < 0.05 (↓)
Wikstrom et al. (2004)	Ankle dorsiflexors and plantarflexors	Peak	Degree	Peak: 0.03 ± 0.03	Peak: 0.03 ± 0.03	Peak: NS: P > 0.05 (↔)
Carcia et al. (2005)	Hip abductors	IC, Peak	Degree	IC: R: −0.19 ± 7.9, L: −0.42 ± 4.7; Peak: R: −10.28 ± 12.4, L: −12.79 ± 9.4	IC: R: −0.93 ± 7.1, L: −1.24 ± 3.9; Peak: R: −9.63 ± 10.9, L: −12.86 ± 9.7	IC: p < 0.05 (↑); Peak: NS: P > 0.05 (R: ↓, L: ↑)
Kim et al. (2021)	Hip abductors	Peak	Degree	Peak: 5.81 ± 6.18	Peak: 5.87 ± 5.42	Peak: NS: P > 0.05 (↑)
Gehring et al. (2008)	Knee flexors and extensors	IC, Peak	Degree	IC: M: 6.7 ± 1.2, F: 3.1 ± 1.1; Peak: M: 6.1 ± 1.3, F: 4.0 ± 3.0	IC: M: 3.5 ± 1.1, F: 6.7 ± 1.1; Peak: M: 5.9 ± 1.3, F: 4.3 ± 3.1	IC: NS: P > 0.05 (R: ↓; L: ↑); Peak: NS: P > 0.05 (↓)
Thomas et al. (2010a)	Hip rotators	IC, Peak	Degree	IC: 4.9 ± 4.2; Peak: 15.6 ± 8.2	IC: 4.2 ± 3.8; Peak: 15.7 ± 8.2	IC: NS: P > 0.05 (↓); Peak: NS: P > 0.05 (↑)
Thomas et al. (2010a)	Ankle plantarflexors	IC, Peak	Degree	IC: 5.0 ± 2.5; Peak: 17.4 ± 5.0	IC: 4.8 ± 2.7; Peak: 17.2 ± 5.3	IC: NS: P > 0.05 (↓); Peak: NS: P > 0.05 (↓)
Knee adduction angle						
Asaeda et al. (2024)	Knee flexors and extensors	Peak vGRF	Degree	Peak vGRF: 20.47 ± 7.433	Peak vGRF: 19.54 ± 9.948	Peak vGRF: NS: P > 0.05 (↓)
Higo and Kuruma (2021)	Knee flexors and extensors	Peak vGRF	Degree	Peak vGRF: 7.1 ± 9.6	Peak vGRF: 6.6 ± 9.1	Peak vGRF: NS: P > 0.05 (↓)
Asaeda et al. (2024)	Hip abductor and adductors	Peak vGRF	Degree	Peak vGRF: 19.29 ± 8.480	Peak vGRF: 21.70 ± 9.150	Peak vGRF: NS: P > 0.05 (↑)
Knee internal rotation angle						
Thomas et al. (2010a)	Knee flexors and extensors	Peak vGRF	Degree	Peak vGRF: M: −0.4 ± 2.3, F: 1.0 ± 4.2	Peak vGRF: M: −2.8 ± 7.1, F: −1.8 ± 3.7	Peak vGRF: p < 0.05 (↑)
Asaeda et al. (2024)	Knee flexors and extensors	Peak vGRF	Degree	Peak vGRF: 4.186 ± 9.978	Peak vGRF:6.281 ± 6.840	Peak vGRF: NS: P > 0.05 (↑)
Asaeda et al. (2024)	Hip abductor and adductors	Peak vGRF	Degree	Peak vGRF: 2.377 ± 11.39	Peak vGRF: 3.345 ± 11.25	Peak vGRF: NS: P > 0.05 (↑)
Kim et al. (2021)	Hip abductors	Peak	Degree	Peak: 8.98 ± 6.93	Peak: 8.15 ± 8.04	Peak: NS: P > 0.05 (↓)
Thomas et al. (2010a)	Hip rotators	IC, Peak	Degree	Peak: 4.6 ± 7.9	Peak: 4.7 ± 8.3	Peak: NS: P > 0.05 (↑)
Thomas et al. (2010a)	Ankle plantarflexors	Peak	Degree	Peak: 2.1 ± 5.4	Peak: 2.1 ± 5.3	Peak: NS: P > 0.05 (↔)
Knee external rotation angle						
Thomas et al. (2010a)	Knee flexors and extensors	IC	Degree	IC: M: −0.5 ± 2.2, F: 1.2 ± 3.4	IC: M: −3.2 ± 7.3, F: −1.5 ± 3.6	IC: p < 0.05 (↑)
Thomas et al. (2010a)	Ankle plantarflexors	IC	Degree	IC: 5.4 ± 4.0	IC: 5.6 ± 3.9;	IC: NS: P > 0.05 (↑);
Thomas et al. (2010a)	Hip rotators	IC	Degree	IC: 3.7 ± 4.7;	IC: 4.9 ± 5.1;	IC: NS: P > 0.05 (↑);
Ankle dorisflexion angle						
Higo and Kuruma (2021)	Knee flexors and extensors	Peak vGRF	Degree	Peak vGRF: 15.2 ± 3.8	Peak vGRF: 15.1 ± 4.5	Peak vGRF: NS: P > 0.05 (↓)
Wikstrom et al. (2004)	Ankle dorsiflexors and plantarflexors	Peak	Degree	Peak: 18.0 ± 10.0	Peak: 18.0 ± 10.0	Peak: NS: P > 0.05 (↔)
Thomas et al. (2010a)	Hip rotators	IC, Peak	Degree	IC: 22.2 ± 14.2; Peak: 14.5 ± 5.7	IC: 24.2 ± 7.3; Peak: 15.1 ± 6.1	IC: NS: P > 0.05 (↑); Peak: NS: P > 0.05 (↑)
Thomas et al. (2010a)	Ankle plantarflexors	IC, Peak	Degree	IC: 23.3 ± 12.1; Peak: 14.5 ± 6.1	IC: 25.3 ± 10.4; Peak: 13.7 ± 6.5	IC: NS: P > 0.05 (↑); Peak: NS: P > 0.05 (↓)
Ankle inversion angle						
Thomas et al. (2010b)	Hip rotators	IC, Peak	Degree	IC: 1.0 ± 6.7;	IC: 1.8 ± 6.9;	IC: NS: P > 0.05 (↑);
Thomas et al. (2010b)	Ankle plantarflexors	IC, Peak	Degree	IC: 1.5 ± 6.1;	IC: 2.0 ± 6.6;	IC: NS: P > 0.05 (↑);
Ankle eversion angle						
Thomas et al. (2010b)	Hip rotators	Peak	Degree	Peak: 8.8 ± 7.5	Peak: 9.5 ± 7.0	Peak: NS: P > 0.05 (↑)
Thomas et al. (2010b)	Ankle plantarflexors	Peak	Degree	Peak: 8.7 ± 5.7	Peak: 8.7 ± 5.2	Peak: NS: P > 0.05 (↔)
Trunk flexion angle						
Higo and Kuruma (2021)	Knee flexors and extensors	Peak vGRF	Degree	Peak vGRF: 13.7 ± 5.9	Peak vGRF: 13.8 ± 5.6	Peak vGRF: NS: P > 0.05 (↑)
Trunk lateral flexion angle						
Kim et al. (2021)	Hip abductors	Peak, Excursion	Degree	Peak: 0.34 ± 6.41,17; Excursion: 34 ± 5.38	Peak: 3.80 ± 6.48; Excursion: 20.40 ± 7.15	Peak: p < 0.05 (↑); Excursion: p < 0.05 (↑)

Abbreviations: M, male; F, female; IC, initial contact; vGRF, vertical Ground Reaction Force; NS, no significant difference; R, right leg; L, left leg; ↑, Parameter changes greater than pre-fatigue; ↔, Parameter changes similar to pre-fatigue; ↓, Parameter changes less than pre-fatigue.

**TABLE 5:** Kinetic variables of the lower limb in landing tasks across various conditions: a synthesis of included studies.

Study	Target areas for fatigue intervention	Phase of kinematic data	Unit of measure	Pre-fatigue	Post-fatigue	Study finding
Hip extension moment						
Thomas et al. (2010a)	Knee flexors and extensors	Peak vGRF	Nm/kg⋅m	Peak vGRF: M: 0.75 ± 0.79, F: 0.74 ± 0.22	Peak vGRF: M:0.77 ± 1.15, F: 0.33 ± 0.74	Peak vGRF: NS: P > 0.05 (M: ↑; F: ↓)
Higo and Kuruma (2021)	Knee flexors and extensors	Peak	Nm/kg	Peak: 0.356 ± 0.286	Peak: 0.299 ± 0.174	Peak: NS: P > 0.05 (↓)
Patrek et al. (2011)	Hip abductors	Peak	Nm	Peak: 41.5 ± 23.1	Peak: 46.1 ± 23.8	Peak: NS: P > 0.05 (↑)
Kim et al. (2021)	Hip abductors	Peak	Nm/kg	Peak: 2.42 ± 0.6	Peak: 2.30 ± 0.41	Peak: NS: P > 0.05 (↓)
Thomas et al. (2010b)	Hip rotators	Peak	Nm/kg	Peak: 0.88 ± 0.59	Peak: 1.07 ± 0.29	Peak: NS: P > 0.05 (↑)
Thomas et al. (2010b)	Ankle plantarflexors	Peak	Nm/kg	Peak: 1.01 ± 0.36	Peak: 1.00 ± 0.31	Peak: NS: P > 0.05 (↓)
Hip abduction moment						
Kim et al. (2021)	Hip abductors	Peak	Nm/kg	Peak: 1.46 ± 0.5	Peak:1.31 ± 0.37	Peak: NS: P > 0.05 (↑)
Thomas et al. (2010b)	Hip rotators	Peak	Nm/kg	Peak: 0.42 ± 0.21	Peak: 0.39 ± 0.26	Peak: NS: P > 0.05 (↓)
Thomas et al. (2010b)	Ankle plantarflexors	Peak	Nm/kg	Peak: 0.31 ± 0.18	Peak: 0.32 ± 0.13	Peak: NS: P > 0.05 (↑)
Hip adduction moment						
Thomas et al. (2010a)	Knee flexors and extensors	Peak vGRF	Nm/kg⋅m	Peak vGRF: M: 0.63 ± 0.32, F: 0.91 ± 0.25	Peak vGRF: M: 0.65 ± 0.84, F: 0.75 ± 0.27	Peak vGRF: NS: P > 0.05 (M: ↑; F: ↓)
Patrek et al. (2011) (Patrek et al., 2011)	Hip abductors	Peak	Nm	Peak: 41.7 ± 20.4	Peak: 31.6 ± 16.7	Peak: p < 0.05 (↓)
Asaeda et al. (2024)	Knee flexors and extensors	Peak vGRF	Nm/kg	Peak vGRF: 0.964 ± 0.282	Peak vGRF: 0.849 ± 0.335	Peak vGRF: NS: P > 0.05 (↓)
Asaeda et al. (2024)	Hip abductor and adductors	Peak vGRF	Nm/kg	Peak vGRF: 0.976 ± 0.386	Peak vGRF: 1.105 ± 0.368	Peak vGRF: NS: P > 0.05 (↑)
Kim et al. (2021)	Hip abductors	Peak	Nm/kg	Peak: 0.02 ± 0.4	Peak: 0.42 ± 0.41	Peak: p < 0.05 (↑)
Hip internal rotation moment						
Thomas et al. (2010a)	Knee flexors and extensors	Peak vGRF	Nm/kg⋅m	Peak vGRF: M: 0.28 ± 0.19, F:0.32 ± 0.15	Peak vGRF: M: 0.30 ± 0.35, F: 0.34 ± 0.17	Peak vGRF: NS: P > 0.05 (↑)
Kim et al. (2021)	Hip abductors	Peak	Nm/kg	Peak: 0.53 ± 0.1	Peak: 0.51 ± 0.17	Peak: NS: P > 0.05 (↓)
Thomas et al. (2010a)	Hip rotators	Peak	Nm/kg	Peak: 0.38 ± 0.10	Peak: 0.39 ± 0.15	Peak: NS: P > 0.05 (↑)
Thomas et al. (2010a)	Ankle plantarflexors	Peak	Nm/kg	Peak: 0.37 ± 0.12	Peak: 0.38 ± 0.12	Peak: NS: P > 0.05 (↑)
Hip external rotation moment						
Kim et al. (2021)	Hip abductors	Peak	Nm/kg	Peak: 0.13 ± 0.1	Peak: 0.21 ± 0.12	Peak: NS: P > 0.05 (↑)
Asaeda et al. (2024)	Knee flexors and extensors	Peak vGRF	Nm/kg	Peak vGRF: 0.352 ± 0.125	Peak vGRF: 0.375 ± 0.125	Peak vGRF: NS: P > 0.05 (↑)
Asaeda et al. (2024)	Hip abductor and adductors	Peak vGRF	Nm/kg	Peak vGRF: 0.339 ± 0.088	Peak vGRF: 0.355 ± 0.088	Peak vGRF: NS: P > 0.05 (↑)
Knee flexion moment						
Thomas et al. (2010a)	Knee flexors and extensors	Peak vGRF	Nm/kg⋅m	Peak vGRF: M: −0.86 ± 0.60, F: −0.74 ± 0.68	Peak vGRF: M: −0.16 ± 0.24, F: −0.43 ± 0.46	Peak vGRF: p < 0.05 (↓)
Knee extension moment						
Higo and Kuruma (2021)	Knee flexors and extensors	Peak	Nm/kg	Peak: 0.088 ± 0.052	Peak: 0.083 ± 0.055	Peak: NS: P > 0.05 (↓)
Patrek et al. (2011)	Hip abductors	Peak	Nm	Peak: 51.0 ± 18.5	Peak: 54.0 ± 19.4	Peak: NS: P > 0.05 (↑)
Kim et al. (2021)	Hip abductors	Peak	Nm/kg	Peak: 2.57 ± 0.3	Peak: 2.30 ± 0.41	Peak: NS: P > 0.05 (↓)
Thomas et al. (2010b)	Hip rotators	Peak	Nm/kg	Peak: 1.67 ± 0.38	Peak: 1.57 ± 0.31	Peak: NS: P > 0.05 (↑)
Thomas et al. (2010b)	Ankle plantarflexors	Peak	Nm/kg	Peak: 1.66 ± 0.35	Peak: 1.64 ± 0.25	Peak: NS: P > 0.05 (↓)
Knee abduction moment						
Kim et al. (2021)	Hip abductors	Peak	Nm/kg	Peak: 0.94 ± 0.4	Peak: 0.79 ± 0.36	Peak: p < 0.05 (↓)
Thomas et al. (2010b)	Hip rotators	Peak	Nm/kg	Peak: 0.82 ± 0.29	Peak: 0.77 ± 0.23	Peak: NS: P > 0.05 (↓)
Thomas et al. (2010b)	Ankle plantarflexors	Peak	Nm/kg	Peak: 0.69 ± 0.48	Peak: 0.75 ± 0.20	Peak: NS: P > 0.05 (↑)
Knee adduction moment						
Thomas et al. (2010a)	Knee flexors and extensors	Peak vGRF	Nm/kg⋅m	Peak vGRF: M: 0.22 ± 0.20, F: 0.09 ± 0.31	Peak vGRF: M: 0.25 ± 0.41, F: 0.14 ± 0.19	Peak vGRF: NS: P > 0.05 (↑)
Patrek et al. (2011)	Hip abductors	Peak	Nm	Peak: 40.2 ± 20.0	Peak: 29.8 ± 17.2	Peak: p < 0.05 (↓)
Asaeda et al. (2024)	Knee flexors and extensors	Peak vGRF	Nm/kg	Peak vGRF: 1.915 ± 0.371	Peak vGRF: 1.249 ± 0.340	Peak vGRF: NS: P > 0.05 (↓)
Asaeda et al. (2024)	Hip abductor and adductors	Peak vGRF	Nm/kg	Peak vGRF: 1.246 ± 0.458	Peak vGRF: 1.409 ± 0.454	Peak vGRF: p < 0.05 (↑)
Kim et al. (2021)	Hip abductors	Peak	Nm/kg	Peak: 0.15 ± 0.4	Peak: 0.42 ± 0.41	Peak: p < 0.05 (↑)
Knee internal rotation moment						
Thomas et al. (2010a)	Knee flexors and extensors	Peak vGRF	Nm/kg⋅m	Peak vGRF: M: −0.11 ± 0.09, F: −0.16 ± 0.14	Peak vGRF: M: −0.04 ± 0.09, F: −0.11 ± 0.12	Peak vGRF: p < 0.05 (↓)
Asaeda et al. (2024)	Knee flexors and extensors	Peak vGRF	Nm/kg	Peak vGRF: 0.191 ± 0.105	Peak vGRF: 0.198 ± 0.129	Peak vGRF: NS: P > 0.05 (↓)
Asaeda et al. (2024)	Hip abductor and adductors	Peak vGRF	Nm/kg	Peak vGRF: 0.226 ± 0.113	Peak vGRF: 0.297 ± 0.145	Peak vGRF: p < 0.05 (↑)
Kim et al. (2021)	Hip abductors	Peak	Nm/kg	Peak: 0.24 ± 0.2	Peak: 0.32 ± 0.30	Peak: p < 0.05 (↑)
Thomas et al. (2010b)	Hip rotators	Peak	Nm/kg	Peak: 0.11 ± 0.07	Peak: 0.11 ± 0.06	Peak: NS: P > 0.05 (↔)
Thomas et al. (2010a)	Ankle plantarflexors	Peak	Nm/kg	Peak: 0.09 ± 0.07	Peak: 0.08 ± 0.05	Peak: NS: P > 0.05 (↓)
Knee external rotation moment						
Kim et al. (2021)	Hip abductors	Peak	Nm/kg	Peak: 0.23 ± 0.19	Peak: 0.22 ± 0.20	Peak: NS: P > 0.05 (↓)
Ankle dorisflexion moment						
Higo and Kuruma (2021)	Knee flexors and extensors	Peak	Nm/kg	Peak: 0.006 ± 0.006	Peak: 0.006 ± 0.010	Peak: NS: P > 0.05 (↔)
Thomas et al. (2010b)	Hip rotators	Peak	Nm/kg	Peak: 1.33 ± 0.26	Peak: 1.25 ± 0.22	Peak: NS: P > 0.05 (↓)
Thomas et al. (2010b)	Ankle plantarflexors	Peak	Nm/kg	Peak: 1.15 ± 0.77	Peak: 1.25 ± 0.20	Peak: NS: P > 0.05 (↑)
Ankle inversion moment						
Thomas et al. (2010b)	Hip rotators	Peak	Nm/kg	Peak: 0.09 ± 0.09	Peak: 0.11 ± 0.06	Peak: NS: P > 0.05 (↑)
Thomas et al. (2010b)	Ankle plantarflexors	Peak	Nm/kg	Peak: 0.10 ± 0.05	Peak: 0.10 ± 0.05	Peak: NS: P > 0.05 (↔)
vGRF						
Higo and Kuruma (2021)	Knee flexors and extensors	Peak	N/kg	Peak: 46.5 ± 9.1	Peak: 45.8 ± 10.7	Peak: NS: P > 0.05 (↓)
Patrek et al. (2011)	Hip abductors	Peak	N	Peak: 1,450 ± 215	Peak: 1,419 ± 231	Peak: NS: P > 0.05 (↓)
Wikstrom et al. (2004)	Ankle dorsiflexors and plantarflexors	Peak	BW	Peak: 3.9 ± 0.70	Peak: 4.2 ± 0.90	Peak: p < 0.05 (↑)
Carcia et al. (2005)	Hip abductors	Peak	BW	Peak: 3.64 ± 0.77	Peak: 3.71 ± 0.76	Peak: NS: P > 0.05 (↑)
Kim et al. (2017)	Knee flexors and extensors	Peak	N/kg	0.3 m: Peak: 26.95 ± 1.88; 0.4 m: Peak: 28.40 ± 1.94	0.3 m: Peak: Post-50%: 26.34 ± 1.92 Peak: Post-30%: 26.61 ± 2.06; 0.4 m: Peak: Post-50%: 27.75 ± 2.22 Post-30%: 28.17 ± 1.78	Peak: p < 0.05 (↓)
Gehring et al. (2008)	Knee flexors and extensors	Peak	BW	Peak: M: 6.2 ± 0.4, F: 5.4 ± 0.4	Peak: M: 6.2 ± 0.5, F: 5.1 ± 0.3	Peak: NS: P > 0.05 (↓)
Kellis and Kouvelioti (2007)	Knee extensors	Peak	BW	Peak: 4.19 ± 0.40	Peak: 3.73 ± 0.47	Peak: p < 0.05 (↓)
Kellis and Kouvelioti (2007)	Knee flexors	Peak	BW	Peak: 4.09 ± 0.41	Peak: 3.98 ± 0.48	Peak: NS: P > 0.05 (↓)
Loading rate						
Higo and Kuruma (2021)	Knee flexors and extensors		N/kg	0.9 ± 0.3	0.9 ± 0.4	NS: P > 0.05 (↔)
Kim et al. (2017)	Knee flexors and extensors		N/kg/s	0.3 m: 211.28 ± 34.36; 0.4 m: 241.11 ± 42.09	0.3 m: Post-50%: 195.98 ± 28.95, Post-30%: 189.50 ± 25.10; 0.4 m: Post-50%: 217.12 ± 32.84, Peak: Post-30%: 220.67 ± 36.20	Peak: p < 0.05 (↓)

Abbreviations: ↑, Parameter changes greater than pre-fatigue; ↔, Parameter changes similar to pre-fatigue; ↓, Parameter changes less than pre-fatigue; M, male; F, female; IC, initial contact; vGRF, vertical Ground Reaction Force; NS, no significant difference; R, right leg; L, left leg; BW, body weight.

### 3.5 Biomechanical outcomes

#### 3.5.1 Hip extensors and knee flexors fatigue protocol

##### 3.5.1.1 Kinematics

In the fatigue protocol involving the hip extensors and knee flexors, only one high-quality study (Klein et al., 2023) investigated the relevant kinematic parameters. The results indicated that, following fatigue, the hip flexion angle at IC, as well as the peak and mean flexion angles, were significantly reduced compared to pre-fatigue levels.

#### 3.5.2 Hip internal and external rotators fatigue protocol

Only one high-quality study examined lower limb kinematic and kinetic parameters during landing tasks following fatigue of the hip internal and external rotators.

##### 3.5.2.1 Kinematics

Thomas et al. (2010b) found that, following fatigue, the hip flexion and internal rotation angles, as well as the peak flexion and internal rotation angles at IC, were greater than those observed pre-fatigue. However, the peak abduction angle at IC was smaller post-fatigue.

Post-fatigue, landing tasks exhibited larger external rotation angles at IC in the knee, as well as greater peak knee flexion, abduction, and internal rotation angles, compared to pre-fatigue. However, the knee’s flexion and abduction angles at IC were reduced post-fatigue.

After fatigue, during landing tasks, the ankle joint showed larger plantarflexion and inversion angles at IC, and larger peak dorsiflexion and eversion angles.

##### 3.5.2.2 Kinetics

The study reported kinetic data only at peak phases. Specifically, it revealed that post-fatigue, there was an increase in the peak flexion and internal rotation moments at the hip joint, the peak extension moment at the knee, and the peak eversion moment at the ankle joint. In contrast, the hip abduction moment, peak knee abduction moment, and peak dorsiflexion moment at the ankle joint were smaller. No significant changes were observed in the peak knee internal rotation moment before and after fatigue.

#### 3.5.3 Hip abductors and adductors fatigue protocol

One high-quality study (Asaeda et al., 2024) reported changes in lower limb biomechanics after a hip abductor and adductor fatigue protocol intervention.

##### 3.5.3.1 Kinematics

This study found that after fatigue, at the peak vGRF moment during landing tasks, the knee exhibited larger inversion and internal rotation angles, while the hip showed smaller abduction and internal rotation angles.

##### 3.5.3.2 Kinetics

After fatigue, at the peak vGRF moment during landing tasks, the hip joint showed larger inversion and external rotation moments, and the knee exhibited larger inversion and internal rotation moments.

#### 3.5.4 Knee extensors fatigue protocol

Only one moderate-quality study (Thomas et al., 2010b) reported changes in lower limb biomechanics before and after the knee extensor fatigue intervention.

##### 3.5.4.1 Kinematics

The kinematic results indicated that after fatigue, the hip flexion angle at IC and the peak flexion angle, as well as the knee flexion angles at both IC and peak flexion, were larger than pre-fatigue.

##### 3.5.4.2 Kinetics

During the landing task under fatigue, the peak vGRF was lower than before fatigue.

#### 3.5.5 Knee flexors fatigue protocol

Only one moderate-quality study (Kellis and Kouvelioti, 2007) reported changes in lower limb biomechanics before and after knee flexor fatigue intervention.

##### 3.5.5.1 Kinematics

The study revealed that, following the fatigue intervention, the angles at IC and peak flexion in both the hip and knee joints were significantly greater compared to pre-fatigue.

##### 3.5.5.2 Kinetics

During the landing task in the fatigued state, the peak vGRF was significantly smaller than before fatigue.

#### 3.5.6 Ankle dorsiflexors and plantarflexors fatigue protocol

Only one moderate-quality study (Wikstrom et al., 2004) reported changes in lower limb biomechanics before and after ankle dorsiflexors and plantarflexors fatigue intervention.

##### 3.5.6.1 Kinematics

Wikstrom et al. (2004) found that, following fatigue, the knee’s peak flexion angle was greater than pre-fatigue. Additionally, no changes were observed in the knee’s peak eversion angle or the ankle’s peak dorsiflexion angle before and after the fatigue intervention.

##### 3.5.6.2 Kinetics

Post-fatigue, the peak vGRF during the landing task was greater than before fatigue.

#### 3.5.7 Ankle plantarflexors fatigue protocol

One high-quality study (Thomas et al., 2010b) reported changes in lower limb biomechanics following the ankle plantarflexors fatigue protocol.

##### 3.5.7.1 Kinematics

This study found that after fatigue, during landing tasks, the following changes were observed in the hip joint: the abduction angle and internal rotation angle at IC, as well as the peak flexion angle, peak abduction angle, and peak internal rotation angle, were all greater than pre-fatigue levels. However, the hip flexion angle at IC was smaller after fatigue compared to before.

In the knee joint, after fatigue, the flexion and abduction angles at IC, as well as the peak flexion and peak abduction angles, were greater than before fatigue. Additionally, the external rotation angle at IC increased, while the peak internal rotation angle remained unchanged.

At the ankle joint, post-fatigue, the plantarflexion and inversion angles at IC were greater than pre-fatigue, while the peak dorsiflexion angle was smaller and the peak eversion angle remained unchanged.

##### 3.5.7.2 Kinetics

The study reported that after fatigue, the hip peak abduction and internal rotation moments were greater than pre-fatigue, while the peak flexion moment was smaller. Post-fatigue, during the landing task at the knee joint, the peak extension and internal rotation moments were smaller than pre-fatigue, whereas the peak abduction moment was significantly greater. Post-fatigue, at the ankle joint, the peak dorsiflexion moment decreased, whereas the inversion moment showed no change.

#### 3.5.8 Hip abductors fatigue protocol

Three high-quality studies (Carcia et al., 2005; Kim et al., 2021; Patrek et al., 2011) reported biomechanical changes in landing tasks following hip abductor fatigue protocol interventions.

##### 3.5.8.1 Kinematics

Patrek et al. (2011) reported that after fatigue, the participants showed reduced flexion angles at the hip joint during IC and 60 ms after IC, while the abduction angle during the same period increased. Another high-quality study showed that, after fatigue, the peak flexion, inversion, and internal rotation angles at the hip were greater than before fatigue.

In a high-quality study (Patrek et al., 2011), the knee flexion angle at IC and the flexion and abduction angles 60 ms after IC were also reported. The results indicated that, post-fatigue, the knee joint exhibited a larger flexion angle at IC, but no significant changes were observed 60 ms after IC. At the same time, the external rotation angle at IC increased after fatigue. Another study (Kim et al., 2021) found that post-fatigue, the peak flexion angle of the knee joint increased, and the internal rotation angle decreased compared to pre-fatigue.

Three high-quality studies (Carcia et al., 2005; Kim et al., 2021; Patrek et al., 2011) simultaneously reported the knee abduction angle during landing tasks. Regarding the knee abduction angle at IC, one study (Carcia et al., 2005) found that the knee abduction angle at IC increased after fatigue, while another study (Patrek et al., 2011) reported a decrease in the knee abduction angle at IC following fatigue. Additionally, two studies (Carcia et al., 2005; Kim et al., 2021) reported the peak knee abduction angle after fatigue, and the results from these studies also showed similar discrepancies.

Finally, one high-quality study (Carcia et al., 2005) reported that, following fatigue, the peak lateral trunk flexion angle and the lateral trunk displacement during landing tasks were increased.

##### 3.5.8.2 Kinetics

Two high-quality studies (Kim et al., 2021; Patrek et al., 2011) concurrently reported the peak extension moment and peak internal rotation moment at the hip joint, as well as the peak extension moment and peak internal rotation moment at the knee joint, but their findings differed. The study by Patrek et al. (2011) reported that, compared to pre-fatigue, post-fatigue there were larger peak hip extension moments, smaller peak hip internal rotation moments, larger peak knee extension moments, and smaller peak knee internal rotation moments. In contrast, the findings of Kim et al. (2021) were opposite to those of Patrek et al. (2011). Furthermore, the peak vGRF results reported by the two studies (Kim et al., 2021; Patrek et al., 2011) were not consistent. The high-quality study by Carcia et al. (2005) found that post-fatigue peak vGRF increased, while Patrek et al. (2011) reported a decrease in peak vGRF after fatigue.

Only the study by Kim et al. (2021) investigated the peak abduction moment, peak internal rotation moment, and peak external rotation moment at the hip joint. The results showed that, after fatigue, the peak hip abduction moment and peak hip internal rotation moment were smaller than pre-fatigue, while the peak hip external rotation moment was also smaller post-fatigue compared to pre-fatigue levels.

Additionally, only one high-quality study by Kim et al. (2021) described the peak valgus moment, peak internal rotation moment, and peak external rotation moment at the knee joint. The study found that, following fatigue, the peak knee valgus moment and peak knee external rotation moment were smaller than pre-fatigue, while the peak knee internal rotation moment was larger post-fatigue compared to pre-fatigue.

#### 3.5.9 Knee flexors and extensors fatigue protocol

A total of three high-quality studies (Asaeda et al., 2024; Kim et al., 2017; Thomas et al., 2010a) and two moderate-quality studies (Gehring et al., 2008; Higo and Kuruma, 2021) reported changes in landing biomechanics before and after the knee flexors and extensors fatigue protocols.

##### 3.5.9.1 Kinematics

One high-quality study (Thomas et al., 2010a) found that post-fatigue, the hip external and internal rotation angles at IC, as well as the external rotation moment at peak vGRF, were greater than pre-fatigue. Additionally, the hip flexion angle at IC was smaller post-fatigue compared to pre-fatigue. Asaeda et al. (2024), in a high-quality study, found that the hip internal rotation angle at peak vGRF was larger post-fatigue. Furthermore, both a high-quality study (Thomas et al., 2010a) and a moderate-quality study (Gehring et al., 2008; Higo and Kuruma, 2021) reported the hip flexion angle at peak vGRF, showing conflicting results. Thomas et al. (2010a) reported that after fatigue, the hip flexion angle at peak vGRF during landing tasks was smaller than before fatigue. However, another study reported the opposite findings. Additionally, both high-quality studies (Asaeda et al., 2024; Thomas et al., 2010a) reported the hip internal rotation angle at peak vGRF, with conflicting results.

Regarding the knee joint, one high-quality study (Thomas et al., 2010a) indicated that after fatigue, the knee’s external rotation angle at IC increased compared to pre-fatigue, while the knee’s abduction angle at peak vGRF decreased. Gehring et al. (2008) found that post-fatigue, the knee’s peak flexion angle was larger than before fatigue, while the knee’s peak abduction angle was smaller. A high-quality study (Asaeda et al., 2024; Thomas et al., 2010a) reported the knee’s external rotation angle at peak vGRF, noting that after fatigue, the knee’s abduction angle at peak vGRF was larger than before fatigue. A moderate-quality study reported the knee’s internal rotation angle at peak vGRF, showing that after fatigue, the internal rotation angle at peak vGRF was larger than pre-fatigue. Higo and Kuruma (2021) and Asaeda et al. (2024) pointed out that after fatigue, the knee’s internal rotation angle at peak vGRF was smaller than before fatigue. Both high-quality studies (Asaeda et al., 2024; Thomas et al., 2010a) found that the knee’s internal rotation angle at peak vGRF was larger before fatigue than post-fatigue. Additionally, discrepancies were observed in the changes in knee flexion and abduction angles at IC between the two studies (Asaeda et al., 2024; Thomas et al., 2010a).

Only one moderate-quality study (Asaeda et al., 2024) reported changes in ankle kinematics before and after the fatigue intervention. This study found that post-fatigue, the ankle’s dorsiflexion angle at peak vGRF was smaller than pre-fatigue.

##### 3.5.9.2 Kinetics

Two high-quality studies (Asaeda et al., 2024; Thomas et al., 2010a) and one moderate-quality study (Gehring et al., 2008) reported that after fatigue, the peak vGRF during landing tasks was smaller than before fatigue. Additionally, both high-quality studies reported the internal rotation moments at the hip and knee joints at peak vGRF. Asaeda et al. (2024), in a high-quality study, found that post-fatigue, both the hip and knee joints exhibited smaller internal rotation moments at peak vGRF compared to pre-fatigue. However, Thomas et al. (2010a) found differing results in male and female participants: post-fatigue, the hip peak internal rotation moment at peak vGRF was greater than pre-fatigue in males, but smaller in females. Similarly, the knee internal rotation moment at peak vGRF was greater after fatigue compared to pre-fatigue.

One moderate-quality study (Gehring et al., 2008; Higo and Kuruma, 2021) reported that post-fatigue, the peak hip extension moment during landing tasks was smaller than before fatigue. Thomas et al. (2010a) reported both the hip extension and internal rotation moments at peak vGRF, showing that after fatigue, the hip extension moment was smaller, while the internal rotation moment was greater than before fatigue. Additionally, Asaeda et al. (2024) found that the hip’s external rotation moment at peak vGRF was larger after fatigue compared to pre-fatigue.

One moderate-quality study (Higo and Kuruma, 2021) reported that post-fatigue, the peak knee extension moment at peak vGRF was smaller than before fatigue. Thomas et al. (2010a) reported that both the knee extension and internal rotation moments at peak vGRF were smaller post-fatigue than pre-fatigue. Asaeda et al. (2024) found that the knee’s external rotation moment at peak vGRF was greater post-fatigue compared to pre-fatigue.

Finally, only one moderate-quality study (Higo and Kuruma, 2021) reported that there was no change in the ankle’s dorsiflexion moment at peak vGRF before and after fatigue.

## 4 Discussion

This review aims to investigate the effects of various peripheral fatigue intervention protocols on the kinematics and kinetics during landing tasks. Nine different peripheral fatigue protocols were included in this review, with only the hip abductors fatigue protocol and knee flexors and extensors fatigue protocol having two or more studies, allowing for evidence synthesis. Among the 14 kinematic parameters reported for these two fatigue protocols, strong evidence indicates that following the knee flexors and extensors fatigue protocol, there is a significant increase in the knee internal rotation angle at peak vGRF. Additionally, moderate evidence suggests a greater knee flexion angle and a smaller knee abduction angle at peak vGRF. Furthermore, within the 13 kinetic parameters reported for these two fatigue protocols, strong evidence indicates that the knee flexors and extensors fatigue protocol leads to a smaller peak vGRF during landing tasks. For the remaining fatigue protocols, the evidence strength for both kinematic and kinetic variables ranges from conflicting to limited, and in some cases, inconclusive (Supplementary Table S3). This highlights the necessity for further research on the different peripheral fatigue intervention protocols in this field.

### 4.1 Hip abductors fatigue protocol

Previous studies have indicated that weakness or fatigue of the hip abductors during tasks that involve deceleration, such as landing, can lead to harmful changes in lower limb biomechanics (Fredericson et al., 2000). This phenomenon is believed to be a key factor contributing to lower limb injuries, particularly ACL injuries (Ireland, 2003; Nadler et al., 2000; Powers, 2003). Further support for the idea that hip abductor weakness increases ACL injury risk during landing tasks has been provided through physical modeling and theoretical perspectives (Chaudhari and Andriacchi, 2005; Powers, 2003; Willson et al., 2005). Weakness in the hip abductors may cause individuals to experience hip adduction or internal rotation during weight-bearing activities, which can indirectly increase the knee abduction angle and moment (Chaudhari and Andriacchi, 2005; Jacobs and Mattacola, 2005). However, the evidence synthesized in this review does not support consistent changes in any knee frontal plane biomechanics pre- and post-fatigue. This is also not consistent with our research hypothesis. Specifically, the evidence for knee abduction angle at IC, peak knee abduction angle, and peak knee adduction moment is conflicting. Prior hip-specific fatigue studies partially explain this conflict. These studies have shown that hip abduction or external rotation forces are weakly or not at all associated with knee kinematics considered harmful to the ACL during landing (Jacobs et al., 2007; Willson and Davis, 2009). This lack of association does not reflect the significant knee abduction predicted by modeling studies. Interestingly, we found conflicting evidence regarding the knee abduction angle at IC. A high-quality study (Carcia et al., 2005) reported that post-fatigue, the knee abduction angle at IC was larger when performing a double-leg landing (p < 0.05), while another high-quality study (Patrek et al., 2011) reported the opposite result, showing a smaller knee abduction angle at IC during a SL task after fatigue (p < 0.05). Furthermore, one high-quality paper (Patrek et al., 2011) reported differing trends in the peak knee abduction angle changes between the left and right legs pre- and post-fatigue. These studies used the same target intervention muscle group but applied different fatigue induction protocols. One study employed repetitive side-lying hip abduction (Patrek et al., 2011), while the other used an isometric bilateral hip abductor fatigue protocol (Carcia et al., 2005). The latter study found that the trends in peak knee abduction angles differed between legs, possibly due to the time discrepancy between the two legs induced by the bilateral fatigue protocol, allowing one leg to recover. This suggests that the intensity and type of fatigue induction protocols may significantly affect lower limb biomechanics during landing. These findings highlight the importance of carefully considering the type of fatigue protocol and its execution when simulating a decline in hip abductor strength, as it can result in considerable variations in biomechanics pre- and post-fatigue.

In this study, the integrated analysis results revealed conflicting evidence regarding peak hip extension moment, peak knee extension moment, and peak vGRF. Larger peak hip extension moment, peak knee extension moment, and peak vGRF are considered to increase lower limb stiffness during landing (Devita and Skelly, 1992). As lower limb stiffness increases, this may lead to greater stress on the ACL, potentially elevating the risk of ACL injury (Hewett et al., 2006). Although these variables did not reach statistical significance (p > 0.05) in the studies, future research needs to further clarify whether this phenomenon is caused by peripheral fatigue effects (or weakness) of the hip abductors, to rule out the potential impact of these variables on ACL injury risk in a fatigued or weakened hip abductor state.

Kim et al. (2021) provided strong evidence indicating that post-fatigue, performing SL tasks resulted in a larger peak hip adduction moment (P < 0.01, Cohen’s d = 0.97), whereas Patrek et al. (2011) found the opposite result. Their study indicated that after a hip abductor fatigue protocol, performing SL tasks led to a smaller peak hip adduction moment (P < 0.01). A systematic review by Barber-Westin and Noyes (2017) also observed a similar phenomenon, finding no consistent evidence indicating that the type of fatigue protocol, the executed task, or the task model (anticipated vs unanticipated) has a strong, consistent impact on lower limb biomechanical changes. To our knowledge, this is the first time a study has summarized the opposite findings in the same muscle-targeted fatigue protocols concerning joint dynamics. The reason for this discrepancy might be that Kim et al.'s fatigue protocol induced hip abductor fatigue through repetitive non-weight-bearing side-lying hip abduction, while Patrek et al. (2011) used an isokinetic dynamometer to induce eccentric contractions of the hip abductors. Different forms of muscle contraction affect muscle fatigue differently, thereby influencing the mechanical performance of the lower limb joints (Zhang et al., 2018). Fatigue protocols using isokinetic contractions, compared to other forms of muscle contraction, usually result in more uniform fatigue, which affects muscle strength output and coordination (Schmitz et al., 2002). Therefore, different muscle contraction forms used in targeted fatigue protocols may induce inconsistent changes in lower limb biomechanics during landing *via* various neuromuscular mechanisms. The optimal use of a hip abductor fatigue protocol to simulate hip abductor weakness remains an open question that warrants further investigation in future studies.

The current evidence remains inconclusive regarding whether hip abductor fatigue or weakness increases the risk of ACL injury during landing. This may be due to the different muscle contraction patterns used in fatigue induction protocols. Future research should continue to explore hip abductor fatigue protocols that mimic the state of hip abductor weakness and investigate whether they increase the risk of ACL injury during landing.

### 4.2 Knee flexors and extensors fatigue protocols

The synergistic action of the knee extensors (quadriceps) and knee flexors (hamstrings) is thought to play a crucial role in reducing the risk of ACL injury during landing tasks by limiting tibial anterior displacement and facilitating energy absorption upon ground contact, thereby forming a muscle-dominant landing strategy (Lloyd and Buchanan, 2001; Thomas et al., 2010a). However, current research has pointed out that the exact effects of simultaneous fatigue or fatigue-induced strength loss in these two muscle groups on ACL injury risk during landing tasks remain unclear (Thomas et al., 2010a). This review partially supports the viewpoints of previous studies, while also revealing some new phenomena: within the same fatigue protocols, conflicting results were observed across different studies regarding the peak hip flexion and internal rotation angles at peak vGRF, knee flexion, and abduction angles at the IC, and hip and knee adduction moments at peak vGRF. Despite these conflicts, consensus was reached on several biomechanical variables. Specifically, following the knee flexors and extensors fatigue protocols, strong evidence indicates that the peak vGRF during landing tasks is reduced compared to pre-fatigue levels. Additionally, moderate evidence suggests that the knee flexion angle at peak vGRF increases, and the knee abduction angle at peak vGRF decreases post-fatigue.

There is still controversy regarding whether fatigue increases lower limb loading during landing tasks. Some studies suggest that after fatigue, individuals land in a more upright and stiff posture, which increases the load on the lower limb (Orishimo and Kremenic, 2007). Conversely, other studies suggest that fatigue activates protective mechanisms, causing the body to significantly increase the range of motion in lower limb joints to reduce the vGRF and alleviate the load on the lower limb, thereby optimizing the landing strategy (Prieske et al., 2017; Zadpoor and Nikooyan, 2012; Zhang et al., 2018) The findings of this review support the latter perspective. We present strong evidence indicating that post-fatigue, executing landing tasks leads to a reduced peak vGRF. Additionally, moderate evidence suggests that the knee flexion angle at peak vGRF increases following fatigue. This suggests that following a knee flexors and extensors fatigue protocol, the lower limb effectively reduces the load by increasing the knee flexion angle and decreasing the peak vGRF during landing tasks.

Lloyd and Buchanan (Lloyd and Buchanan, 2001), using an electromyography-based knee biomechanics model, estimated the muscle contribution to external moments and found that the co-contraction of the quadriceps and hamstrings acts as the primary stabilizer of the knee in the frontal plane, particularly during landing and deceleration tasks. However, with the onset of fatigue, the strength of these two muscle groups decreases, compromising knee stability in the frontal plane and potentially reducing overall knee stability. Our review findings are consistent with this observation, as we found moderate evidence suggesting that fatigue results in a smaller knee abduction angle at peak vGRF during landing tasks. This change may reflect an adaptive response of the knee to fatigue, where athletes are unable to maintain their original movement patterns, leading to adjustments in the landing strategy (Gehring et al., 2008). Furthermore, we found strong evidence showing that after fatigue, the knee internal rotation angle at peak vGRF increases during landing. Previous studies have indicated that a reduction or imbalance in quadriceps and hamstring strength can lead to excessive knee internal rotation. This study found that changes in the frontal and horizontal planes of the knee after fatigue are unlikely to be potential risk factors for ACL injury. However, existing literature indicates that excessive knee motion in the frontal and horizontal planes during landing significantly increases the risk of ACL injury., with a higher susceptibility to injury compared to mechanisms induced by sagittal plane motion (Hewett et al., 2005; McLean et al., 2004; Trigsted et al., 2017). Our review also revealed conflicting evidence regarding the knee abduction angle at IC, as well as the knee adduction moment at IC and peak vGRF, following the knee flexor and extensor fatigue protocols during landing tasks. These parameters are considered critical indicators for ACL injury (McLean et al., 2004; Olsen et al., 2004; Quatman and Hewett, 2009), and the conflicting results may be attributed to differences in fatigue induction protocols, such as variations in speed settings (120°/s vs 180°/s) on isokinetic devices and the different forms of fatigue induction (isokinetic vs repetitive fixed-load protocols). Different muscle contraction methods may lead to inconsistent effects on lower limb biomechanics following fatigue during landing. Therefore, future research should focus on clarifying the biomechanical changes in the knee frontal and horizontal planes during landing tasks, specifically regarding the effects of fatigue in the knee flexors and extensors muscle groups, and further explore the relationship between these changes and ACL injury.

### 4.3 Other fatigue protocols

In this study, three muscle-specific fatigue intervention protocols were identified that had a significant impact (p < 0.05) on the biomechanics of the corresponding joint or segment. However, no such significant effects were observed in other peripheral fatigue protocols. Thomas et al. proposed that this phenomenon may be influenced by the length of the lever arm between the targeted muscle group and the joint it controls (Thomas et al., 2010a). For instance, the quadriceps both extend the knee and flex the hip. In the horizontal plane, the quadriceps have a very small lever arm at the hip, whereas at the knee joint, the lever arm is relatively large. Consequently, quadriceps fatigue or weakness has a greater effect on knee biomechanics than on hip biomechanics. This phenomenon was also observed in the knee extensors and flexors fatigue protocols in our study. We found that after executing these two fatigue protocols, significant changes in knee flexion angle were observed during landing tasks (P < 0.05) compared to pre-fatigue conditions, while no changes were found in hip biomechanics.

The results of this review suggest that peripheral fatigue interventions targeting the muscles responsible for sagittal plane movements at the knee joint (e.g., knee extensors and knee flexors fatigue protocols) may activate protective mechanisms in the lower limbs. Specifically, this is manifested by an increase in the knee flexion angle at IC, peak knee flexion angle (P < 0.05), and peak hip flexion angle (P < 0.05), along with a reduction in peak vGRF (P < 0.05), which helps reduce the load on the lower limbs and, in turn, lowers the risk of injury (Alentorn-Geli et al., 2009). During landing tasks, the knee extensors and flexors usually work in co-contraction to stabilize the joint and decelerate the body. However, when one of these muscle groups becomes fatigued or weakened, the imbalance between the two muscle groups leads to a reduction in joint control, increasing joint instability (Gioda et al., 2024). To counteract this instability, the body may increase knee flexion to help distribute the load, reduce the impact force on the joint, and decrease the risk of injury. It is worth noting that previous peripheral fatigue interventions targeting the sagittal plane muscle groups of the knee have often been used to simulate quadriceps weakness in patients with ACL injuries or those who have undergone reconstruction (Song et al., 2024). These interventions have been employed to study movements such as single-leg hopping and single-leg landing, with the aim of developing new clinical assessments of lower limb strength. However, this study found that peripheral fatigue interventions targeting the sagittal plane muscle groups of the knee may activate protective mechanisms in the lower limbs, which could lead to discrepancies in the results of previous studies that used healthy individuals to simulate ACL injuries and reconstruction outcomes through peripheral fatigue induction.

This review found that the fatigue protocols for ankle plantarflexors and dorsiflexors resulted in a greater peak vGRF during landing (p < 0.05). The ankle plantarflexor fatigue protocol was also associated with a smaller knee flexion angle at IC (p < 0.05). These variables are considered biomechanical factors that are linked to the risk of ACL injury. He et al., in their systematic review and meta-analysis comparing individuals with chronic ankle instability and healthy controls during landing tasks, observed similar phenomena to those found in this review. Specifically, chronic ankle instability patients showed greater peak vGRF and smaller hip flexion angles during landing (He et al., 2024). During landing tasks, the ankle joint is the first lower limb joint to be influenced by the ground reaction forces. The ankle plays a crucial role in maintaining postural stability and balance during the landing phase (Lee and Lin, 2007). Additionally, in the case of functionally abnormal ankle joints, compensatory movement patterns often occur at proximal joints (Brown et al., 2004; Caulfield and Garrett, 2002). This compensation could potentially contribute to ACL injury risk (Padua et al., 2009; Trigsted et al., 2017).

This review briefly summarizes the overall trends in lower limb biomechanical changes before and after fatigue for each protocol. While most fatigue protocols demonstrated changes in joint angles and moments post-fatigue, the limited number of studies for each protocol has resulted in generally low evidence strength. Therefore, it remains challenging to draw definitive conclusions from the available data. Future research should aim to conduct more experiments involving peripheral fatigue interventions to fill the current research gaps and clarify the relationship between different isolated fatigue protocols and ACL injury risk. This will help provide more concrete evidence on how peripheral muscle fatigue, particularly in isolated muscle groups, influences lower limb biomechanics and ACL injury mechanisms.

### 4.4 Limitations

Although this review provides a comprehensive systematic evaluation of the biomechanical parameters related to ACL injury risk during landing tasks following peripheral fatigue, several limitations should be acknowledged: First, in this review, we defined the fatigued muscles as those actively involved in the joint movements targeted by the fatigue protocol. However, it is important to note that for multi-joint muscles, such as the quadriceps, this definition may introduce ambiguity. While our approach is consistent with how fatigue protocols are typically induced based on joint movements, the induction of fatigue in single muscle groups has yet to be standardized. From a kinematic perspective, this differentiation of targeted muscle groups appears to be a reasonable approach (Delp et al., 1994; Pressel and Lengsfeld, 1998). Additionally, the review focused solely on landing tasks, which may not fully capture the biomechanical changes associated with other dynamic movements like cutting or sudden stops (Giesche et al., 2021). Furthermore, all studies included tasks performed under predictable conditions, limiting the applicability to real-world, unpredictable scenarios. The variety of landing tasks included also makes it difficult to generalize findings, suggesting that future research should define specific tasks and muscle groups more clearly. The demographic of participants—mostly young, recreational athletes—limits generalizability to other populations, such as elite athletes or older adults. Lastly, the review did not sufficiently differentiate between peripheral and central fatigue, which could influence the biomechanical outcomes and warrants further investigation to understand their distinct contributions.

## 5 Conclusion

### 5.1 Summary

Our findings suggest that following the knee flexor and extensor fatigue protocol, there is strong evidence of an increase in the knee internal rotation angle at peak vGRF and a decrease in peak vGRF during landing tasks. Additionally, when implementing the hip abductors fatigue protocol, conflicting evidence was observed regarding the changes in the peak hip adduction moment (p < 0.01) during landing tasks. These mixed results and the significant conflict in some of the biomechanical variables indicate that further research is needed in this area. Future studies should focus on improving peripheral fatigue protocols and methods for measuring fatigue. Such improvements will help clarify whether current neuromuscular ACL injury prevention programs need to be adapted to account for the biomechanical changes brought about by peripheral fatigue.

### 5.2 Practical implications

Over the past three decades, numerous studies have explored lower limb biomechanical changes induced by muscle-specific fatigue, attempting to unveil the mechanisms of lower limb injuries (Patrek et al., 2011), develop new injury prevention tests (Song et al., 2024), and support physical therapy and clinical practice. However, this review highlights that even when targeting the same muscle group, different fatigue protocols can lead to discrepancies, or even significant variations, in landing biomechanics. These inconsistencies may be attributed to differences in the types of muscle contractions used in the fatigue protocols, as well as the varying thresholds for measuring fatigue. Therefore, this review suggests that peripheral fatigue tasks induced during different sports activities are not identical. As such, injury prevention programs should be tailored to the specific characteristics of each sport, with fatigue prevention training designed accordingly to reduce the risk of injury. Future research should focus on the standardization of fatigue protocols. A more systematic investigation into the relationship between different forms of peripheral fatigue and joint injuries—particularly concerning muscle-induced inhibition and strength loss following injury—is necessary. Additionally, advanced technologies such as high-density EMG, magnetic resonance imaging, and musculoskeletal ultrasound should be employed to conduct deeper mechanical analyses of the immediate biomechanical effects of fatigue protocols that utilize various muscle contraction types. This approach could offer a more precise understanding of how fatigue-induced changes in muscle performance impact joint stability and injury risk, paving the way for more effective injury prevention strategies.

## Data Availability

The original contributions presented in the study are included in the article/Supplementary Material, further inquiries can be directed to the corresponding author.
