# Supplementary material for: Effects of different peripheral fatigue protocol on lower limb biomechanical changes during landing and its impact on the risk of anterior cruciate ligament injury: a systematic review
Source: Front Bioeng Biotechnol. 2025 Jun 3;13:1587573. doi: 10.3389/fbioe.2025.1587573 (PMC12188353; doi:10.3389/fbioe.2025.1587573)
Supplement: Supplementary file 1 [file Table1.docx]

Supplementary Material

# Supplement Table 1. Search Strategy

**Web of Science Search Strategy**

(((TS=(stop jump OR stop-jump OR stop jumping OR stop-jumping OR land OR landing OR jump land OR jump landing OR jump-land OR jump-landing OR Drop landing OR drop-vertical jump OR single-leg landing OR single-leg land OR unilateral land OR jump OR jumping)) AND TS=(kinematic OR kinetics OR biomechanics OR Moment OR Torque OR dynamic)) AND TS=(Leg OR lower limb OR lower extremity OR hip OR knee OR ankle)) AND TS=(fatigue OR fatigability OR exertional OR Exhaustion OR exhausted OR depletion)= 727

**Pubmed Search Strategy**

(((stop jump OR stop-jump OR stop jumping OR stop-jumping OR land OR landing OR jump land OR jump landing OR jump-land OR jump-landing OR Drop landing OR drop-vertical jump OR single-leg landing OR single-leg land OR unilateral land OR jump OR jumping) AND (kinematic OR kinetics OR biomechanics OR Moment OR Torque OR dynamic)) AND (Leg OR lower limb OR lower extremity OR hip OR knee OR ankle)) AND (fatigue OR fatigability OR exertional OR Exhaustion OR exhausted OR depletion)= 508

**Scopus Search Strategy**

( TITLE-ABS-KEY ( "stop jump" OR "stop-jump" OR "stop jumping" OR "stop-jumping" OR "land" OR "landing" OR "jump land" OR "jump landing" OR "jump-land" OR "jump-landing" OR "Drop landing" OR "drop-vertical jump" OR "single-leg landing" OR "single-leg land" OR "unilateral land" OR "jump" OR "jumping" ) AND TITLE-ABS-KEY ("kinematic" OR "kinetics" OR "biomechanics" OR "Moment" OR "Torque" OR "dynamic" ) AND TITLE-ABS-KEY ( "Leg" OR "lower limb" OR "lower extremity" OR "hip" OR "knee" OR "ankle" ) AND TITLE-ABS-KEY ( "fatigue" OR "fatigability" OR "exertional" OR "Exhaustion" OR "exhausted" OR "depletion" ) ) =492

**EBSCO Search Strategy**

TI ( stop jump OR stop-jump OR stop jumping OR stop-jumping OR land OR landing OR jump land OR jump landing OR jump-land OR jump-landing OR Drop landing OR drop-vertical jump OR single-leg landing OR single-leg land OR unilateral land OR jump OR jumping ) AND TI ( kinematic OR kinetics OR biomechanics OR Moment OR Torque OR dynamic ) AND TI ( Leg OR lower limb OR lower extremity OR hip OR knee OR ankle ) AND TI ( fatigue OR fatigability OR exertional OR Exhaustion OR exhausted OR depletion ) = 83

AB ( stop jump OR stop-jump OR stop jumping OR stop-jumping OR land OR landing OR jump land OR jump landing OR jump-land OR jump-landing OR Drop landing OR drop-vertical jump OR single-leg landing OR single-leg land OR unilateral land OR jump OR jumping ) AND AB ( kinematic OR kinetics OR biomechanics OR Moment OR Torque OR dynamic ) AND AB ( Leg OR lower limb OR lower extremity OR hip OR knee OR ankle ) AND AB ( fatigue OR fatigability OR exertional OR Exhaustion OR exhausted OR depletion )= 896

**Cochrane Library Search Strategy**

stop jump OR stop-jump OR stop jumping OR stop-jumping OR land OR landing OR jump land OR jump landing OR jump-land OR jump-landing OR Drop landing OR drop-vertical jump OR single-leg landing OR single-leg land OR unilateral land OR jump OR jumping in Title Abstract Keyword AND kinematic OR kinetics OR biomechanics OR Moment OR Torque OR dynamic in Title Abstract Keyword AND Leg OR lower limb OR lower extremity OR hip OR knee OR ankle in Title Abstract Keyword AND fatigue OR fatigability OR exertional OR Exhaustion OR exhausted OR depletion in Title Abstract Keyword = 195

# Supplement Table 2. Downs and Black checklist quality assessment criteria

| **Question** | **Rating criteria** |
| --- | --- |
| **Reporting** | |
| 1. Is the hypothesis/aim/objective of the study clearly described? | 1 The aim of the study is clearly described in the Introduction section  0 No information is provided |
| 2. Are the main outcomes to be measured clearly described in the Introduction or Methods sections? | 1 If the outcomes to be measured are mentioned in the Methods section.  0 If this information is mentioned in the Results section first. |
| 3. Are the characteristics of the subjects included in the study clearly described? | 1 If the inclusion/exclusion criteria for the study is provided  0 If information such as age, height and weight are provided but inclusion/exclusion criteria not mentioned. |
| 5. Are the distributions of principle confounders in each group of subjects to be compared clearly described? | 1 Fatigue level is provided  0 No information is provided |
| 6. Are the main findings of the study clearly described? | 1 If the outcome data was clearly defined  0 If there were discrepancies in determining the outcome |
| 7. Does the study provide estimates of the random variability in the data for the main outcome? | 1 If studies have provided Standard deviation, 95%CI or Standard error values  0 If no such information is provided |
| 10. Have actual probability values been reported (e.g. 0.035 rather than < 0.05) for the main outcomes except where the probability is less than 0.001? | 1 If exact p-value for a particular outcome is provided.  0 If this information is not available. |
| **External validity** | |
| 11. Were the subjects asked to participate in the study representative to the entire population from which they were recruited? | 1 If the population was females with no restriction on the sport they participated (2 or more)  0 If the population was females from on specific sport |
| 12. Were those subjects who were prepared to participate representative of the entire population from which they were recruited? | 1 If the number of participants who refused was stated in the study  0 No information is provided |
| **Internal validity** | |
| 15- Was an attempt made to blind those measuring the main outcome? | 1 If an attempt was made to blind the people conducting the experiment  0 No information was provided |
| 16- If any of the results was based on “data dredging”, was this made clear? | 1 Any analyses that had not been planned at the outset of the study should be clearly indicated  0 No |
| 18. Were the statistical tests used to assess the main outcomes appropriate? | 1 The statistics used in the study were appropriate for analysing the data set  0 No |
| 20. Were the main outcome measures used accurate (valid and reliable)? | 2 Accuracy and methods are reported  1 Only Methods are reported  0 No such information |
| **Internal validity- Confounding (risk of bias)** | |
| 21. Were the subjects (e.g. the two groups to be compared) recruited from the same population? | 1 If they were from the same clinic or school etc.  0 If they were from different locations or no information was provided |
| 22. Were the study subjects (the two groups to be compared) recruited over the same period of time? | 1 If this information was provided in the study  0 Studies that does not specify the time period over which patients were recruited |
| 25. Were there adequate adjustments for confounding in the analyses from which the main findings were drawn? | 1 If the effect of the main confounders was investigated or confounding was demonstrated  0 If the effect of the main confounders was not investigated or confounding was demonstrated but no adjustment was made in the final analysis. |
| **Power analysis** | |
| 27. Did the study have sufficient power to detect a clinically important effect? | 1 Performed power or sample size analysis  0 No information is provided |

# Supplement 3: Strength of evidence for the kinematic and kinetic variables reported in the included studies

| **Target areas for fatigue intervention** |  | | | | | | **Study findings** |
| --- | --- | --- | --- | --- | --- | --- | --- |
| **Hip extensors and knee flexors** | **Kinematics parameter** | | | **Overall studies** | **Quality rating of the relevant studies** | **Strength of evidence** |  |
|  | Joint | Phase | Joint Motion |  |  | Pre- vs. Post- |  |
|  | Hip | IC | Flexion | 1 |  | Limited | ↓ |
|  |  | Peak | Flexion | 1 |  | Limited | ↓ |
|  |  | Mean | Flexion | 1 |  | Limited | ↓ |
| **Hip abductors** | **Kinematics parameter** | | | **Overall studies** | **Quality rating of the relevant studies** | **Strength of evidence** |  |
|  | Joint | Phase | Joint Motion |  |  | Pre- vs. Post- |  |
|  | Hip | IC | Flexion | 1 |  | Limited | ↓ |
|  |  |  | Abduction | 1 |  | Limited | ↑ |
|  |  | Peak | Flexion | 1 |  | Limited | ↑ |
|  |  |  | Adduction | 1 |  | Limited | ↑ |
|  |  |  | Internal Rotation | 1 |  | Limited | ↑ |
|  |  | at 60 ms | Flexion | 1 |  | Limited | ↓ |
|  |  |  | Abduction | 1 |  | Limited | ↑ |
|  | Knee | IC | Flexion | 2 | 1,1 | Limited | ↑ |
|  |  |  | Abduction | 2 | 1,1 | Conflicting | ↑,↓ |
|  |  | Peak | Flexion | 2 | 1,1 | Limited | ↑ |
|  |  |  | Abduction | 2 | 1,1 | Conflicting | ↑,↓ |
|  |  |  | Internal Rotation | 1 |  | Limited | ↓ |
|  |  | at 60 ms | Flexion | 1 |  | Limited | -- |
|  |  |  | Abduction | 1 |  | Limited | ↑ |
|  | Trunk | Peak | Lateral Flexion | 1 |  | Limited | ↑ |
|  |  | Excursion | Lateral Flexion | 1 |  | Limited | ↑ |
|  | **Kinetics parameter** | | | **Overall studies** | **Quality rating of the relevant studies** | **Strength of evidence** |  |
|  | Joint | Phase | Joint Motion |  |  | Pre- vs. Post- |  |
|  | Hip | Peak | Extension | 2 | 1,1 | Conflicting | ↑,↓ |
|  |  |  | Adduction | 2 | 1,1 | Conflicting | ↑,↓ |
|  |  |  | Abduction | 1 |  | Limited | ↓ |
|  |  |  | Internal Rotation | 1 |  | Limited | ↓ |
|  |  |  | External Rotation | 1 |  | Limited | ↑ |
|  | Knee | Peak | Extension | 2 | 1,1 | Conflicting | ↑,↓ |
|  |  |  | Adduction | 2 | 1,1 | Conflicting | ↑,↓ |
|  |  |  | Abduction | 1 |  | Limited | ↓ |
|  |  |  | Internal Rotation | 1 |  | Limited | ↑ |
|  |  |  | External Rotation | 2 | 1, | Limited | ↓ |
|  | Impact loading | Peak | VGRF |  |  | Conflicting | ↑,↓ |
| **Hip rotators** | **Kinematics parameter** | | | **Overall studies** | **Quality rating of the relevant studies** | **Strength of evidence** |  |
|  | Joint | Phase | Joint Motion |  |  | Pre- vs. Post- |  |
|  | Hip | IC | Flexion | 1 |  | Limited | ↑ |
|  |  |  | Abduction | 1 |  | Limited | ↓ |
|  |  |  | Internal Rotation | 1 |  | Limited | ↑ |
|  |  | Peak | Flexion | 1 |  | Limited | ↑ |
|  |  |  | Abduction | 1 |  | Limited | ↓ |
|  |  |  | Internal Rotation | 1 |  | Limited | ↑ |
|  | Knee | IC | Flexion | 1 |  | Limited | ↓ |
|  |  |  | Abduction | 1 |  | Limited | ↓ |
|  |  |  | External Rotation | 1 |  | Limited | ↑ |
|  |  | Peak | Flexion | 1 |  | Limited | ↑ |
|  |  |  | Abduction | 1 |  | Limited | ↑ |
|  |  |  | Internal Rotation | 1 |  | Limited | ↑ |
|  | Ankle | IC | Plantaflexion | 1 |  | Limited | ↑ |
|  |  |  | Inversion | 1 |  | Limited | ↑ |
|  |  | Peak | Dorsiflexion | 1 |  | Limited | ↑ |
|  |  |  | Eversion | 1 |  | Limited | ↑ |
|  | **Kinetics parameter** | | | **Overall studies** | **Quality rating of the relevant studies** | **Strength of evidence** |  |
|  | Joint | Phase | Joint Motion |  |  | Pre- vs. Post- |  |
|  | Hip | Peak | Flexion | 1 |  | Limited | ↑ |
|  |  |  | Abduction | 1 |  | Limited | ↓ |
|  |  |  | Internal Rotation | 1 |  | Limited | ↑ |
|  | Knee | Peak | Extension | 1 |  | Limited | ↑ |
|  |  |  | Abduction | 1 |  | Limited | ↓ |
|  |  |  | Internal Rotation | 1 |  | Limited | -- |
|  | Ankle | Peak | Dorsiflexion | 1 |  | Limited | ↓ |
|  |  |  | Eversion | 1 |  | Limited | ↑ |
| **Hip abductor and adductors** | **Kinematics parameter** | | | **Overall studies** | **Quality rating of the relevant studies** | **Strength of evidence** |  |
|  | Joint | Phase | Joint Motion |  |  | Pre- vs. Post- |  |
|  | Hip | Peak vGRF | Abduction | 1 |  | Limited | ↓ |
|  |  |  | Internal rotation | 1 |  | Limited | ↓ |
|  | Knee | Peak vGRF | Adduction | 1 |  | Limited | ↑ |
|  |  |  | Internal rotation | 1 |  | Limited | ↑ |
|  | **Kinetics parameter** | | | **Overall studies** | **Quality rating of the relevant studies** | **Strength of evidence** |  |
|  | Joint | Phase | Joint Motion |  |  | Pre- vs. Post- |  |
|  | Hip | Peak vGRF | Adduction | 1 |  | Limited | ↑ |
|  |  |  | Internal rotation | 1 |  | Limited | ↑ |
|  | Knee | Peak vGRF | Adduction | 1 |  | Limited | ↑ |
|  |  |  | Internal rotation | 1 |  | Limited | ↑ |
| **Knee flexors and extensors** | **Kinematics parameter** | | | **Overall studies** | **Quality rating of the relevant studies** | **Strength of evidence** |  |
|  | Joint | Phase | Joint Motion |  |  | Pre- vs. Post- |  |
|  | Hip | IC | Flexion | 1 |  | Limited | ↓ |
|  |  |  | Abduction | 1 |  | Limited | ↑ |
|  |  |  | Internal Rotation | 1 |  | Limited | ↑ |
|  |  | Peak vGRF | Flexion | 2 | 1,2 | Conflicting | ↑↓ |
|  |  |  | Abduction | 2 | 1,1 | Limited | ↑ |
|  |  |  | Adduction |  |  | Limited | ↑ |
|  |  |  | Internal Rotation | 2 | 1,1 | Conflicting | ↑↓ |
|  | Knee | IC | Flexion | 2 | 1,2 | Conflicting | ↑↓ |
|  |  |  | Abduction | 2 | 1,2 | Conflicting | ↓↑ |
|  |  |  | External Rotation | 1 |  | Limited | ↑ |
|  |  | Peak | Flexion | 1 |  | Limited | ↑ |
|  |  |  | Abduction | 1 |  | Limited | ↓ |
|  |  | Peak vGRF | Flexion | 2 | 1,2 | Moderate | ↑ |
|  |  |  | Abduction | 1 |  | Limited | ↓ |
|  |  |  | Adduction | 2 | 2,1 | Moderate | ↓ |
|  |  |  | Internal Rotation | 2 | 1,1 | Strong | ↑ |
|  | Ankle | Peak vGRF | Dorsiflexion | 1 |  | Very limited | ↓ |
|  | Trunk | Peak vGRF | Flexion | 1 |  | Very limited | ↑ |
|  | **Kinetics parameter** | | | **Overall studies** | **Quality rating of the relevant studies** | **Strength of evidence** |  |
|  | Joint | Phase | Joint Motion |  |  | Pre- vs. Post- |  |
|  | Hip | Peak | Extension | 1 |  | Very limited | ↓ |
|  |  | Peak vGRF | Extension | 1 |  | Limited | ↓ |
|  |  |  | Adduction | 2 | 1,1 | Conflicting | ↓↑ |
|  |  |  | Internal Rotation | 1 |  | Limited | ↑ |
|  |  |  | External Rotation | 1 |  | Limited | ↑ |
|  | Knee | Peak | Extension | 1 |  | Very limited | ↓ |
|  |  | Peak vGRF | Flexion | 1 |  | Limited | ↓ |
|  |  |  | Adduction | 2 | 1,1 | Conflicting | ↑↓ |
|  |  |  | Internal Rotation | 1 |  | Limited | ↑ |
|  |  |  | External Rotation | 1 |  | Limited | ↓ |
|  | Ankle | Peak vGRF | Dorsiflexion | 1 |  | Very limited | -- |
|  | Impact loading | Peak | vGRF | 3 | 2,1,1 | Strong | ↓ |
|  |  |  | Loading Rate | 1 |  | Very limited | -- |
| **Knee extensors** | **Kinematics parameter** | | | **Overall studies** | **Quality rating of the relevant studies** | **Strength of evidence** |  |
|  | Joint | Phase | Joint Motion |  |  | Pre- vs. Post- |  |
|  | Hip | IC | Flexion | 1 |  | Very Limited | ↑ |
|  |  | Peak | Flexion | 1 |  | Very Limited | ↑ |
|  | Knee | IC | Flexion | 1 |  | Very Limited | ↑ |
|  |  | Peak | Flexion | 1 |  | Very Limited | ↑ |
|  | **Kinetics parameter** | | | **Overall studies** | **Quality rating of the relevant studies** | **Strength of evidence** |  |
|  | Joint | Phase | Joint Motion |  |  | Pre- vs. Post- |  |
|  | Impact Loading | Peak | vGRF | 1 |  | Very Limited | ↓ |
| **Knee flexors** | **Kinematics parameter** | | | **Overall studies** | **Quality rating of the relevant studies** | **Strength of evidence** |  |
|  | Joint | Phase | Joint Motion |  |  | Pre- vs. Post- |  |
|  | Hip | IC | Flexion | 1 |  | Very Limited | ↑ |
|  |  | Peak | Flexion | 1 |  | Very Limited | ↑ |
|  | Knee | IC | Flexion | 1 |  | Very Limited | ↑ |
|  |  | Peak | Flexion | 1 |  | Very Limited | ↑ |
|  | **Kinetics parameter** | | | **Overall studies** | **Quality rating of the relevant studies** | **Strength of evidence** |  |
|  | Joint | Phase | Joint Motion |  |  | Pre- vs. Post- |  |
|  | Impact Loading | Peak | vGRF | 1 |  | Very Limited | ↓ |
| **Ankle dorsiflexors and plantarflexors** | **Kinematics parameter** | | | **Overall studies** | **Quality rating of the relevant studies** | **Strength of evidence** |  |
|  | Joint | Phase | Joint Motion |  |  | Pre- vs. Post- |  |
|  | Knee | Peak | Flexion | 1 |  | Very Limited | ↑ |
|  |  |  | Abduction | 1 |  | Very Limited | -- |
|  | Ankle | Peak | Dorsiflexion | 1 |  | Very Limited | -- |
|  | **Kinetics parameter** | | | **Overall studies** | **Quality rating of the relevant studies** | **Strength of evidence** |  |
|  | Joint | Phase | Joint Motion |  |  | Pre- vs. Post- |  |
|  | Impact Loading | Peak | vGRF | 1 |  | Very Limited | ↑ |
| **Ankle plantarflexors** | **Kinematics parameter** | | | **Overall studies** | **Quality rating of the relevant studies** | **Strength of evidence** |  |
|  | Joint | Phase | Joint Motion |  |  | Pre- vs. Post- |  |
|  | Hip | IC | Flexion | 1 |  | Limited | ↓ |
|  |  |  | Abduction | 1 |  | Limited | ↑ |
|  |  |  | Internal Rotation | 1 |  | Limited | ↑ |
|  |  | Peak | Flexion | 1 |  | Limited | ↑ |
|  |  |  | Abduction | 1 |  | Limited | ↑ |
|  |  |  | Internal Rotation | 1 |  | Limited | ↑ |
|  | Knee | IC | Flexion | 1 |  | Limited | ↓ |
|  |  |  | Abduction | 1 |  | Limited | ↓ |
|  |  |  | External Rotation | 1 |  | Limited | ↑ |
|  |  | Peak | Flexion | 1 |  | Limited | ↓ |
|  |  |  | Abduction | 1 |  | Limited | ↓ |
|  |  |  | Internal Rotation | 1 |  | Limited | -- |
|  | Ankle | IC | Plantarflexion | 1 |  | Limited | ↑ |
|  |  |  | Inversion | 1 |  | Limited | ↑ |
|  |  | Peak | Dorsiflexion | 1 |  | Limited | ↓ |
|  |  |  | Eversion | 1 |  | Limited | -- |
|  | **Kinetics parameter** | | | **Overall studies** | **Quality rating of the relevant studies** | **Strength of evidence** |  |
|  | Joint | Phase | Joint Motion |  |  | Pre- vs. Post- |  |
|  | Hip | Peak | Flexion | 1 |  | Limited | ↓ |
|  |  |  | Abduction | 1 |  | Limited | ↑ |
|  |  |  | Internal Rotation | 1 |  | Limited | ↑ |
|  | Knee | Peak | Extension | 1 |  | Limited | ↓ |
|  |  |  | Abduction | 1 |  | Limited | ↑ |
|  |  |  | Internal Rotation | 1 |  | Limited | ↓ |
|  | Ankle | Peak | Dorsiflexion | 1 |  | Limited | ↑ |
|  |  |  | Inversion | 1 |  | Limited | -- |

Green circle: High quality study; Origan circle: Moderate quality study
